# Occurrence and Characteristics of Mobile Colistin Resistance (*mcr*) Gene-Containing Isolates from the Environment: A Review

**DOI:** 10.3390/ijerph17031028

**Published:** 2020-02-06

**Authors:** Madubuike Umunna Anyanwu, Ishmael Festus Jaja, Obichukwu Chisom Nwobi

**Affiliations:** 1 Microbiology Unit, Department of Veterinary Pathology and Microbiology, University of Nigeria, Nsukka 400001, Nigeria; madubuike.anyanwu@unn.edu.ng; 2 Department of Livestock and Pasture Science, University of Fort Hare, Alice 5700, South Africa; 3 Department of Veterinary Public Health and Preventive Medicine, University of Nigeria, Nsukka 400001, Nigeria; obichukwu.nwobi@unn.edu.ng

**Keywords:** plasmid-mediated, *mcr* gene, environment, antimicrobial resistance

## Abstract

The emergence and spread of mobile colistin (COL) resistance (*mcr*) genes jeopardize the efficacy of COL, a last resort antibiotic for treating deadly infections. COL has been used in livestock for decades globally. Bacteria have mobilized *mcr* genes (*mcr*-1 to *mcr*-9). Mcr-gene-containing bacteria (MGCB) have disseminated by horizontal/lateral transfer into diverse ecosystems, including aquatic, soil, botanical, wildlife, animal environment, and public places. The *mcr*-1, *mcr*-2, *mcr*-3, *mcr*-5, *mcr*-7, and *mcr*-8 have been detected in isolates from and/or directly in environmental samples. These genes are harboured by *Escherichia coli*, *Enterobacter*, *Klebsiella*, *Proteus*, *Salmonella*, *Citrobacter*, *Pseudomonas*, *Acinetobacter*, *Kluyvera*, *Aeromonas*, *Providencia*, and *Raulotella* isolates. Different conjugative and non-conjugative plasmids form the backbones for *mcr* in these isolates, but *mcr* have also been integrated into the chromosome of some strains. Insertion sequences (IS) (especially IS*Apl1*) located upstream or downstream of *mcr*, class 1–3 integrons, and transposons are other drivers of *mcr* in the environment. Genes encoding multi-/extensive-drug resistance and virulence are often co-located with *mcr* on plasmids in environmental isolates. Transmission of *mcr* to/among environmental strains is clonally unrestricted. Contact with the *mcr*-containing reservoirs, consumption of contaminated animal-/plant-based foods or water, international animal-/plant-based food trades and travel, are routes for transmission of MGCB.

## 1. Introduction

The emergence and spread of plasmid-mediated movable colistin (COL) resistance (*mcr*) genes jeopardize the efficacy of COL considered a last resort drugs for treating deadly infections caused by multi- and extensively drug-resistant Gram-negative bacilli (GNB) [1,2]. All the while, COL resistance was thought to be due to chromosomal mutations (such as widely dispersed two-component system *prmAB* and *PhoPQ*, and *mgBr* in *Klebsiella*), which are not transferable [3]. However, in 2015, *mcr*-1 gene was detected in *Escherichia coli* isolates from humans, food animals and the environment in China [4]. Indeed, this heralded the emergence of pandrug-resistant bacteria (superbugs) [5]. Since, after the discovery of *mcr*-1 gene, eight other *mcr* gene types (*mcr*-2 to *mcr*-9) with their very many variants have been detected in isolates from humans, animals, and environment in six of the seven continents (except for Antarctica) [6,7,8,9]. The *mcr* genes encode MCR, which are cytoplasmic transmembrane proteins found in GNB [10]. These proteins are phosphoethanolamine (pEtN) transferases conferring resistance to COL by attaching a pEtN moiety to the lipid A of lipopolysaccharide in bacterial cell membrane thereby abolishing the negative charges to which cationic colistin/polymyxins have affinity [3,10,11,12]. Because COL was barely used in human medicine from 1970 to 1994 [13] but has been habitually used for livestock production for more than 50 years, it is clear that COL selection pressure evolved due to veterinary use [14,15]. There is an avalanche of publications in the literature showing that worldwide, humans and animals (especially livestock) are colonized by commensal and potentially pathogenic colistin-resistant organisms (COLROS)/*mcr* gene-containing bacteria (MGCB) [16,17,18]. The rapid global spread of MGCB has left the world with a very limited therapeutic option such as tigecycline [19]; but unfortunately, plasmid-mediated mobile genes encoding high tigecycline level resistance was recently detected [20]. By this development, the global public health is gravely threatened, and the spread of these superbugs continues. Worryingly, the O’Neill’s projection that, if the cause of antimicrobial resistance (AMR) is not tackled now, 10 million human lives could be lost worldwide annually by 2050 due to antimicrobial-resistant infections [21] might become a reality.

It is largely recognized that the environment plays a huge role in disseminating clinically relevant antimicrobial resistance genes (ARGs) [22,23,24,25]. Accordingly, the United Nations Environment Programme (UNEP) recently identified environmental AMR as the top six emerging issues of concern [25,26]. In One Health model, surveillance of ARGs in humans, animals, soil, plant, aquatic and aquaculture environment is crucial in determining the extent of dissemination of a targeted ARG [2,27]. Potentially pathogenic COLROS emanating from any of these ecosystems can move with relative ease to another without barriers [2,25]. Experts agree that the control of ARGs’ spread cannot be achieved without tackling this problem from the environment [2,22,23,24,25,26]. It is established that antimicrobial selection pressure in the environment results due to discharges from humans and animals (containing un-metabolized antimicrobials in faeces/urine into sewage), hospitals, industries, and run-offs from refuse dumps (often contains anthropogenic wastes) and farmlands especially where biocides are used [11,12,23,24,25,28]. Putative sources of MGCB in the environment include human and animal excretions, aquaculture water, sewages/wastewaters from hospitals and laboratories [11,12,23,28]. The hospital environment often contains numerous antibiotics which perhaps stimulate COL resistance [29]. Thus hospital effluents constitute a putative source of COLROS in water and soil ecosystem [30]. The most worrisome situation about COL resistance is the presence of other antimicrobial agents (including disinfectants and metals) in an environment, could stimulate COL selection pressure, and *mcr* gene is often co-located with multiple AMR (most troubling being carbapenemases, extended-spectrum β-lactamases [ESBL], and plasmid-mediated quinolone resistance [PMQR] encoding genes) and virulence genes on highly promiscuous plasmids [31]. There have been extensive discourse on acquired resistance [32,33,34,35] and virulence genes [36], as well as the drivers (plasmids, integrons, transposons, insertion sequences, etc.) facilitating the horizontal/lateral spread (by conjugation, transformation or transduction) of these genes among bacterial population [37,38,39,40,41,42]. Of the drivers of resistance, plasmids are the major vehicle involved in the transmission of resistance/virulence genes [39,40,41,42]. Plasmids are small circular/linear double-stranded DNA unit which is chromosome independent and capable of self-replication, and they belong to many families (with many incompatibilities and replicons) with broad or narrow host ranges [37,41,43]. Resistance and virulence plasmids carry genes encoding antimicrobial resistance and virulence factors, respectively, thus enhancing bacterial survival fitness [38,43].

The use of antimicrobials as biocides on plants potentially results in colonization of plants and soil by antimicrobial-resistant organisms [43]. Use of animal manure and insufficiently-treated/untreated sewage sludge as fertilizer in farmlands and aquaculture are other potential routes of introduction of COLROS into the soil and aquaculture [43,44]. Plants (fruits, vegetables, and grasses) could get contaminated with COLROS emanating from soil [45,46]. Unfortunately, these fruits and crops are often consumed raw or undercooked by humans and animals thus posing a risk to public health [46]. Run-offs from farmlands primarily where insufficiently-treated wastewater, sewage sludge or animal manure was used as fertilizer, and aquaculture water discharged or used for irrigation, may introduce COLROS/*mcr* genes and un-metabolized antimicrobials into aquatic systems (surface/groundwater bodies and aquaculture) in which bacteriophages (by transduction) and integrons could facilitate the spread of *mcr* gene [11,23,24,29,30]. In fact, evidence has shown that *mcr* and non-mobile COL resistance (*nmcr*) genes were mobilized from perhaps aquatic environment [2,27,47,48,49]. Feral aquatic animals (such as fish, birds, mammals, reptiles, amphibians, molluscs, etc) could get colonized by COLROS, thereby contaminating the food chain [23,50].

The role of wildlife (especially those that migrate for long distances) in the dissemination of clinically-relevant ARGs and the public health impact is well recognized [50,51,52,53,54]. Although antimicrobials are not used in wildlife, contact with human and domesticated animal excretions (especially for terrestrial animals) in the environment, could result in infection of wildlife by COLROS [52,53]. Consumers of undercooked wildlife meat products are at huge risk of acquisition of COLROS [50,55]. Carnivores could also get colonized by MGCB through feeding on wild animals, and then further disseminate *mcr* genes in the environment. Moreover, wild animals could disseminate *mcr* genes to places (such as playing grounds, farmlands, parks, markets, open slaughterhouses) frequented by people [50,52,55]. Syanthropic flies that feed on garbage, carrion, human and animal wastes could get colonized by COLROS. Often the colonization of fly occurs on their body surfaces or intestines where the horizontal exchange of ARGs takes place. Thereby serving as reservoirs or vectors of transmission of these organisms to other ecological niches when they perch on animals, plants, foods and contact surfaces in households and public places, and/or when they serve as feed for fish and birds [56,57,58]. Wild birds (especially migratory birds) could deposit MGCB (through defecation, grooming or drinking) into surface waters and onto fruits/vegetables [45,59]. In some parts of the world (especially in rural areas in developing countries), surface water is used for bathing, laundering, recreation, fishing, and as drinking water for human and animals [60,61,62]. Such activities could facilitate the exchange of COLROS from the environment to humans and animals [60,62]. Integrated farms facilitate the exchange of COLROS between food animals and aquaculture since excretions from livestock that may contain COLROS/MGCB and un-metabolized COL, serve as food to fish which receives little or no supplementation [23,24,28,48]. Farmworkers’ paraphernalia (such as gloves, boots, wheel-barrow, vehicles, etc.) are potential vehicles of transmission of COLROS from farm-yards to other ecological niches [63].

Presence of *mcr* genes in the natural environment could complicate the transmission dynamics of COLROS, thus impacting the epidemiology and increasing the rate of evolution of MGCB [64,65]. Understanding the occurrence and magnitude of COL resistance in the environment creates the needed impetus to tackle the problem [66]. Information on the occurrence, phenotypic and genotypic characteristics of MGCB isolated from the environment is crucial for an understanding of the epidemiology, genetic environment and mechanism of acquisition of *mcr* genes by environmental isolates. Such information would be necessary in designing and prioritizing surveillance programs that may generate essential data for performing risk assessment, implementation of effective antimicrobial stewardship plans, developing effective strategies for control of COLROS, and reducing the risk to public health. In this review, the objective is to report the findings of studies on plasmid-mediated COL resistance among isolates from the environment.

## 2. Literature Search Strategy and Data Extraction

Studies that assessed the presence of *mcr* genes in isolates from environmental sources (water, sewages, aquaculture, aquatic-based foods, wildlife including flies and reptiles) worldwide were included in this review. Peer-reviewed works of literature were identified by searching databases such as Pubmed, MEDLINE, EMBASE, Scopus, and Web of knowledge. Google search engine was also used to retrieve grey literature. The following search terms and/or text words were used for the search: “mobile colistin resistance gene”, “plasmid-mediated colistin resistance gene”, “plasmid-borne COL resistance”, “mobile COL resistance”, “movable COL resistance genes”, “enterobacteria”, “Gram-negative bacilli”, “bacterial isolates”, “environment”, “wildlife”, “wild animals”, “migratory animals”, soil”, “water”, sewages”, “fish”, “aquatic-based foods”, and names of specific countries in the world. References of identified publications were reviewed for additional pertinent articles. Information extracted from included studies includes the first author’s surname, year of publication, and country where the study was conducted. Other information extracted were isolation/study period, type of *mcr* gene assayed, sample processed, number of samples, number of isolates subjected to *mcr* assay. We also extracted the number and type of organism positive for *mcr* gene, *mcr* gene variant detected; sequence type, virulence genes, plasmid type, associated insertion sequence, and additional resistance factors identified in test isolate (Table 1, Table 2, Table 3, Table 4, Table 5, Table 6, Table 7 and Table 8).

## 3. Plasmid-Mediated Colistin Resistance in Isolates from Environmental Ecosystems

### 3.1. Environmental Contact Surfaces

The presence of antimicrobial-resistant organisms on environmental contact surfaces is concerning because these surfaces play a role in the epidemics of COLROS [67]. Seven publications investigated on plasmid-mediated colistin resistance in a total of 775 isolates from environmental contact surfaces. Two of the studies investigated the presence of *mcr* gene directly in the samples. Fifty-four isolates (33 *E. coli*, 8 Klebsiellae, 4 *Acinetobacter iwofii*, 6 *Enterobacter*, and 1 each for *Citrobacter freundii*, *Pseudomonas aeruginosa*, and *Proteus putida*) were reported to harbour *mcr*-1 among the tested isolates.

In Asia, contact surfaces in livestock farms were reported to be reservoirs of MGCB. Two *mcr*-1-carrying nonpathogenic strains were detected among 9 *E. coli* isolates from fences at a pig farm in China [68], suggesting that animals potentially contaminate their immediate environment with COLROS. Animal dejections (urine and faeces), wild animals (rodents, insects), contaminated water and feed, and/or hands of persons visiting/working in the farm are potential sources/routes of contamination of contact surfaces in a farm environment. In the *mcr*-1-positive *E. coli* strains, *mcr*-1 was located on plasmids of various sizes (<60–150 kb) suggesting that the gene could be transferred between plasmids resulting in its rapid spread among bacterial population [68]. In another study from the same country, 26 *mcr*-1-carrying enterobacterial strains (23 *E. coli* and 3 *K. pneumoniae*) were isolated from contact surfaces (hand rails, vending machine and so on) at public transportation routes [67], suggesting that these surfaces are sources of colonization of travelers by COLROS and that travelers potentially disseminate these organisms from one location to another thereby posing public health risks [69]. Interestingly, most of the *mcr*-1-positive strains were recovered from samples collected from areas with a high density of hospitals or traffic indicating that these isolates could be of nosocomial origin thus highlighting the need for hand hygiene to prevent transmission of MGCB capable of causing diseases with pandemic potential. The *mcr*-1 was located on different plasmids (IncI2, IncX4 and IncH2) and the *mcr*-1-positive *E. coli* isolates belonged to phylogroups B1 and A, suggesting that diverse promiscuous plasmids facilitate the spread of *mcr*-1 among commensal *E. coli* strains in the environment. There were 4 resistance genes (including ESBL gene) in 2 different antibiotic families in the *mcr*-1-positive *E. coli* isolates and the *K. pneumoniae* isolates, indicating that diverse multidrug-resistant organisms (MDROS) could be acquired during travel and subsequently disseminated to other locations thus posing serious public health risks [69]. The *mcr*-1-positive *E. coli* isolates were extensively diverse (belonging to 9 different STs) with pandemic high-risk (HiR) international zoonotic clones ST10 (the most dominant ST complex of human strains) and ST101 complexes [70,71] being predominant while all the *K. pneumoniae* isolates were of ST37 (Table 1). Thus, suggesting that hands of travelers are routes for disseminating hospital-acquired colistin-resistant enterobacterial clones into the public. This could result in the outbreak of hard-to-treat pandemic infections that can easily spread among individuals in densely-populated areas as often seen at transportation routes. Presence of MGCB on environmental contact surfaces in other Asian countries is yet to be reported. However, it is worth noting that in Bangladesh, *mcr*-1 and *mcr*-2 were detected in *E. coli* isolates from poultry birds/street foods but not in samples from poultry farm environment [72], suggesting that COLROS may not survive harsh environmental conditions particularly where high biosecurity measures (such as disinfection of farm environment) are put in place.

**Table 1:** Studies reporting plasmid-mediated colistin resistance in isolates from environmental contact surfaces.

Country	Source of Isolate	Date of Isolation (*mcr* Gene Assayed)	Number of Isolates Tested for *mcr*	Identified Gene/Variant (Number of Organism)	Sequence Type and/or Phylogroup (Virulence Genes)	Plasmid (Associated Insertion Sequence)	Additional Resistance Traits	Key Points/Conclusion	Reference
Italy	Hospital surfaces	2016–2017 (*mcr*-1)	300	*mcr*-1 (4 *Acinetobacter iwoffi* and *E. coli*, *Citrobacter freundii* (1), *Pseudomonas aeruginosa* (1) and *Proteus putida* (1), *Enterobacter (E.) cloacae* (3) and *E. agglomerans* (3), 6 *K. pneumoniae* and 2 *K. oxytoca*)	-	-	-	- *mcr*-1-carrying GNB contaminate surfaces in hospitals representing a reservoir of hard-to-treat nosocomial pathogens	[29]
China	Contact surfaces at public transportation routes	2016–2017 (*mcr*-1)	*mcr* was detected in samples	*mcr*-1 (23 *E. coli* and 3 *K. pneumoniae*)	*E. coli* (ST2253, ST58, ST48, ST1249, ST7122, ST744, ST189, ST101, and ST10 complex); *K. pneumoniae* (ST37)	IncI2, IncX4 and IncH2.	-	- First report of *mcr*-1-carrying organisms contaminating contact surfaces at public transportation routes in China	[67]

*mcr*: mobile colistin resistance gene; -: no data; Additional resistance traits: Additional resistance traits: resistance factors identified in one *mcr*-positive isolate or pooled factors in more than one *mcr*-positive isolate; Sequence type: comprise all sequence types of *mcr* gene-positive isolates; Plasmid: plasmid types identified in a/pooled *mcr* gene-positive isolate; Inc.: incompatibility; IS: insertion sequence.

Farm worker’s paraphernalia were reported to be vehicles for transmission of MGCB in Europe. Two strains carrying *mcr*-1 on IncX4 plasmid were detected among 25 *E. coli* isolates (8%) from boots used 50–150 m distance from pig farms in Germany [65], suggesting that farmers’ wears (often in contact with animal dejections) are potential vehicles for transmission of MGCB from livestock farms to other locations. Thus contact with materials used in animal farms is a potential route for the acquisition of COLROS. In the strains, *mcr*-1 was carried on plasmids of varied sizes, further suggesting that *mcr*-1 is transferred between plasmids thereby facilitating its rapid spread among bacterial population. The *mcr*-1-positive *E. coli* strains were of ST1140 and HiR ExPEC clone ST10 that causes intestinal and several extraintestinal diseases worldwide [70,71,73], and harboured 10 other resistance genes (including ESBL gene) in 5 different antimicrobial families (Table 8) suggesting they are multidrug-resistant organisms (MDROS) capable of causing hard-to-treat infections thereby posing a serious challenge to public health. It was also reported that the ST10 isolate and another *mcr*-1-IncX4-positive faecal *E. coli* isolate from a barn dog were related differing only by 7 single nucleotide polymorphisms (SNPs) suggesting that IncX4 is a major driver of *mcr*-1 among *E. coli* strains colonizing different ecological niches [65]. In the same country, a retrospective survey detected 43 *mcr*-1-carrying strains among 436 pooled faecal/boot swab *E. coli* isolates (9.9%) from 15 farms [74], further indicating that farmers or their wears are vehicles for transmission of COLROS. Contact surfaces in European hospitals were also reported to be reservoirs of MGCB. Twenty-five *mcr*-1-carrying multidrug-resistant (MDR) strains (4 each of *Acinetobacter iwoffi*, and *E. coli*, 1 each of *Citrobacter freundii*, *Pseudomonas aeruginosa* and *Proteus putida*, 3 each of *Enterobacter (E.) cloacae* and *E. agglomerans*, 6 *K. pneumoniae*, and 2 *K. oxytoca*) were detected among 300 isolates (8.3%) from hospital surfaces in Italy [29], suggesting that these surfaces represent huge reservoir of diverse hard-to-treat nosocomial pathogens that can easily diffuse into the public thereby portending danger to public health. Intriguingly, a *Proteus putida* strain harboured *mcr*-1 thereby suggesting that the ability of *Proteus* species to acquire/transfer *mcr* have been underestimated all this while that this organism was regarded as being intrinsically-resistant to COL hence a non-*mcr*-carrier [75,76,77].

### 3.2. Sewages/Wastewaters

Wastewaters and sewages contain nutrients that support the growth of bacteria, thus are increasingly being recognized as a source of new emerging pathogens and antibiotic resistance [78]. The more concern about wastewater treatment plants (WWTP) is the hygienic quality of receiving waters and for water reuse [78]. Fourteen publications investigated the *mcr* gene in a total of 185 isolates from sewages/wastewaters. Sixty-three isolates (57 *E. coli*, 3 *Kluyvera*, 2 *Klebsiella pneumoniae* and 1 unspecified isolate) were reported to harbour *mcr*-1 among the isolates tested. Four of the studies investigated *mcr* gene directly in sewage/wastewater samples.

In Europe, wastewaters and sewages are noted as reservoirs of *mcr*-1. In a German study, *mcr*-1 was detected in influent and effluent waters of 3 out of 7 WWTPs [79], indicating that WWTPs are reservoirs of COLROS. Remarkably, one of the WWTPs containing *mcr*-1 had never received wastewater from hospitals, intensive animal farms or food industry, thus suggesting that domestic wastewater (from toilets and bathrooms) or wildlife (rodents, insects, birds, amphibians, worms, and so on) might be the sources of the gene in those plants. Apart from *mcr*-1, other resistance genes (including ESBL and AmpC genes) in 3 different antibiotic classes were detected in the wastewaters (Table 2), suggesting that WWTPs are reservoirs of multiple resistance genes originating from anthropogenic sources. These WWTPs are sources from which *mcr*/other ARGs are spreading to other ecosystems. Although *mcr*-1-carrying bacteria were not recovered from the wastewater samples, gene markers for *A. baumanii*, *E. coli* and *K. pneumoniae* were detected, thus incriminating these organisms as reservoirs of *mcr*-1 in wastewaters. In a recent study from the same country, a relatively lower copy number of *mcr*-1 compared to *mcr*-3, *mcr*-4, *mcr*-5 and *mcr*-7 was detected in 14 municipal wastewater samples [80], suggesting that wastewaters from anthropogenic sources are cocktails of *mcr* genes and potential sources of dissemination of these determinants to other ecosystems. Therefore surveillance of only an *mcr* gene-type in an environmental matrix could result in underestimation of the magnitude of this global problem [80]. Thus, in order to adequately determine the extent of plasmid-mediated colistin resistance in an ecological niche, there is the need for assaying different *mcr* gene-types thus warranting the development of affordable rapid kits that could detect all the existing *mcr* gene-types and the ones that are yet to emerge.

In a study from Spain, 30 strains (29 *E. coli* and one *K. pneumoniae*) carrying *mcr*-1 on IncI2 plasmid were detected among 90 isolates (33.3%) from sewage [81], suggesting that IncI2 plasmid is one of the common drivers of *mcr*-1 in the sewage environment. There was diversity among the isolates with the ESBL-producing MPEC strains belonging to ST1196 and ST224 while the *K. pneumoniae* strain belonged to ST526 (Table 2). This means that diverse multidrug-resistant (MDR) COLROS are present in sewage in Spain. Though the ST224 strain harboured *mcr*-1, it exhibited COL susceptibility which might be caused by the cell wall structure or the copy number of *mcr*-1-IncI2 plasmid [81]. This suggests that *mcr*-1 could act as a silent gene evading phenotypic detection, thereby favouring its dissemination [81]. Transformation of *mcr*-1 from the *mcr*-1-positive *E. coli* strains into a recipient organism was positive, indicating that these strains can rapidly spread the gene to other organisms by HGT [82]. In another Spanish study, *mcr*-1 was detected in filtered pellets from untreated and treated wastewater of domestic, hospital, industrial and agricultural origins [66], further indicating that these are sources of COLROS in WWTPs. Expectedly, the untreated wastewater contained a significantly higher *mcr*-1 gene copy numbers than treated wastewater meaning that treating wastewater decreases the number of COLROS but does not entirely remove the *mcr*-1. Thus, even after treatment, wastewater may still be a source of *mcr*-1 into receiving water bodies [66]. This warrants the need for improvement of protocols used in sewage/wastewater treatment.

The presence of MGCB in wastewater/sewages/sludges in Asian countries has been reported. Two pathogenic strains carrying *mcr*-1 on IncHI2 and IncX4 plasmids were detected among 65 ESBL-producing *E. coli* isolates (3.1%) from canal water in Thailand [83], suggesting that diverse plasmids are spreading genes conferring COL resistance and resistance against last-resort antibiotics in the study area. In a Bangladeshi study, an *mcr*-1-carrying *E. coli* strain was detected among 48 isolates from sludge samples [84], suggesting that *mcr*-1-positive *E. coli* has dispersed into the Bangladesh environment possibly due to improper disposal of anthropogenic/agricultural wastes.

In China, several reports indicated that sewages/wastewaters constitute reservoirs of COLROS/*mcr* genes. Eighteen of 24 samples of wastewater at different stages of treatment (75%), including water to be released to the sea post-treatment were observed to contain *mcr*-1-carrying *E. coli* and *Klebsiella* strains [85], further indicating that COLROS can survive during wastewater treatment thereby ending up in water bodies. This highlights the need for effective sewage/wastewater treatment methods. A surveillance study on dissemination of *mcr* and carbapenem resistance in Chinese poultry production sector detected *mcr*-1 in sewage sample from a poultry farm, and an enterobacterial isolate carrying *mcr*-1 on IncI plasmid was obtained from the sample [86], further suggesting that IncI plasmid is a major driver of COL resistance in livestock environment and that animal farm wastes constitute a reservoir for MGCB. In another Chinese study, 11 *mcr*-1carrying strains (9 *E. coli* and 2 *Kluyvera*) were detected in sewage samples [87]. Two of the *mcr*-1-positive *E. coli* strains harboured new *mcr*-1 variants, named *mcr*-1.7 on IncI2 plasmid and *mcr*-1.4 on IncX4 plasmid; further indicating that IncI2 and IncX4 plasmids are the commonest drivers of *mcr* in uncommon and common species from the environmental ecosystem in China. This may mirror what is happening in human/animal ecosystems in the country since the sewage samples originated from these settings. Apart from the fact that the *mcr*-1-positive *E. coli* strains transferred *mcr*-1 genes at a high frequency (10^−5^ to 10^−7^ cells per recipient cell) to a recipient organism, the presence of IS*ApI1* upstream of *mcr*-1 in the strains imply they could rapidly transfer the genes to diverse organisms. However, *mcr*-1 was chromosomally-located in 2 of the *mcr*-1-positive *E. coli* isolates indicating that these organisms maintain the gene through the vertical transmission to their progenies hence making elimination of the gene from the environment practically impossible.

Furthermore, there were 31 other resistance genes (including ESBL and pAmpC genes) in 8 different antimicrobial families in the isolates (Table 2), further indicating that the *mcr*-1-positive *E. coli* strains were multidrug-resistant organisms (MDROS) thus posing challenge to public health. Nevertheless, the strains exhibited susceptibility to some antimicrobial agents, including imipenem and tigecycline. It is however troubling that the MPEC strains were extensively diverse (5 STs) with some isolates belonging to zoonotic HiR ExPEC clones (ST10, ST34, ST48). This further suggests that transmission of *mcr*-1 among environmental *E. coli* strains is nonclonal.

Several Chinese studies noted that hospital sewage (which may contain clinical specimens such as faeces, fluid, blood, decomposed tissues and so on) is a potential source/route of spread of COLROS. In a study on the global spread of *mcr*-1, 8 *mcr*-1-carrying *E. coli* strains were recovered from influents/effluents of tertiary care teaching hospitals in China [2]. In another study from China, a multidrug-resistant ST313 *K. pneumoniae* strain carrying *mcr*-1on IncP-1 plasmid (a broad host-range incompatibility group) was isolated from hospital sewage [88], suggesting that sewages are sources for the dissemination of *mcr*-1 plasmids transferrable to diverse organisms. There was IS*Apl1* downstream of *mcr*-1 suggesting that the gene was acquired, and 9 other resistance genes in 9 different antimicrobial families as well as a novel ORFble gene with yet unknown function (Table 2), further suggesting that sewages in China are potential sinks for novel antimicrobial determinants [88]. In another Chinese study, 9 *mcr*-1-carrying *E. coli* strains were detected among 25 *Enterobacteriaceae* (isolated from hospital sewage) [89], suggesting that *E. coli* is the most common COLROS in sewage ecosystem. In the *mcr*-1-positive *E. coli* isolates, *mcr*-1 including 4 genes encoding β-lactam resistance was located on ~33 kb IncX4 plasmid (Table 2). There was extensive diversity among the strains (7STs) some of which were zoonotic HiR ExPEC lineages such as ST10 and ST101 [70,71]. These *E. coli* clones might eventually be released into farmlands and water bodies. Interestingly, an *mcr*-1-IncX4 plasmid of the ST10 and ST7122 isolates conjugated with the genome of a recipient organism at very high-frequency rates of 2.79 × 10^7^–7.60 × 10^8^, further indicating that IncX4 is a major driver of COL resistance in environmental strains.

From the same country, a *Kluyvera ascobarta* strain carrying *mcr*-1 on IncI2 plasmid was recovered from hospital sewage [90], further indicating that diverse uncommon species are spreading COL and multiple resistance genes in the Chinese environment. The *Kluyvera* strain can rapidly spread COL resistance having transferred *mcr*-1-IncI2 plasmid to a recipient organism at a very high frequency (10^−5^ cells per recipient). There were 2 other resistance genes (including a novel ESBL gene) conferring resistance to 2 different antimicrobial classes (multi-resistance) in the strain with extensive susceptibility to 14 antimicrobials, including imipenem, implying that *mcr*-1 is not necessarily carried by extensively drug-resistant strains [90]. In a recent Chinese study, an *E. coli* ST410/phylogroup A strain carrying a rare combination of *mcr*-1.1, *mcr*-3.5 and *bla*_NDM-5_ on a 265.5 kb IncHI2-ST3 plasmid (containing IncN, IncP and IncX3 plasmids) was isolated from influx mainstream of hospital sewage [9], further indicating that HiR zoonotic ExPEC clones [91] are circulating *mcr*-1 on diverse plasmids in the Chinese environment. It is worrisome that the organism harboured 31 other resistance genes in 10 different antimicrobial families meaning it is extensively drug-resistant; treatment of infections caused by extensively drug-resistant strains are often difficult. However, the organisms were susceptible to aztreonam-avibactam and tigecycline. A similar amikacin-susceptible clinical human ST206 *E. coli* strain also harbouring *mcr*-1.1, *mcr*-3.5 and *bla*_NDM-5_ was also detected in China [92], suggesting that diverse *E. coli* clones containing COL and carbapenem resistance genes, are circulating in human and environmental ecosystems in the country. However, the sewage and human isolates were not the same since the human strain did not contain *rmt* gene which codified high-level amikacin resistance; nevertheless, there is the possibility of acquisition of this gene from the sewage environment. Unfortunately, *E. coli* clones containing both carbapenem and COL resistance genes have disseminated into the environmental ecosystem; these determinants can rapidly spread to diverse clones [9,92,93,94]. Worse of it, is that the *mcr*-1.1-, *mcr*-3.5-, *bla*_NDM-5_- and *rmt*-plasmids, were transferred to another organism at a very high (10^−3^–10^−4^) frequency. In fact, these 4 genes together were transferred at an outrageously high (10^−10^) frequency, suggesting that multiple drug resistance genes, even when not located on the same plasmid can be spread at a very high rate [9]. 

The spread of MGCB into aquatic ecosystem due to poor environmental sanitation has been reported in Asia. Eight *mcr*-1-carrying multidrug-resistant *Proteus mirabilis* strains were isolated from sewer waters in the Syrian war refugee camps in Lebanon [95], further suggesting that *Proteus* species are potentially *mcr*-harbouring organisms which should no longer be neglected. Retrospective studies to investigate *mcr* in COL-R *Proteus* isolates from various ecosystems are warranted. There was a β-lactam determinant as well as class 1 integrons in the strains, indicating that diverse MGEs facilitate the spread of *mcr*-1 in aquatic ecosystems. Interestingly, the *Proteus* strains expressed biofilm meaning they can potentially persist in the camp environment making it practically impossible to eliminate *mcr*-1. This finding of *mcr*-1-positive *Proteus* in refugee camps calls for urgent intervention because these organisms can cause highly-resistant clinical conditions (such as bacteraemia, urinary, respiratory, eye, and wound infections) mostly affecting immunocompromised persons (such as children, women and diseased individuals) predominating in the camps [95]. 

**Figure 1:**
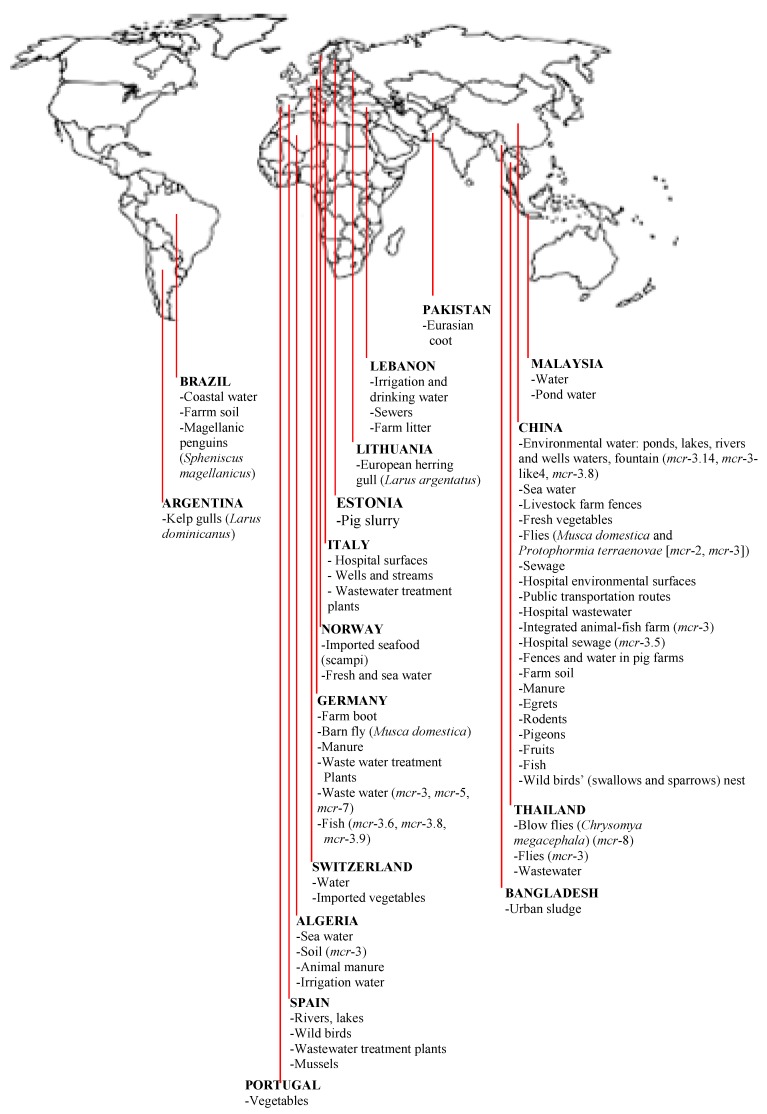
Countries in which mobile colistin resistance (*mcr*) gene *mcr*-1, including other *mcr* gene types indicated, have been detected in environmental (water, aquaculture, soil, plant, sewage, wastewater, and wildlife) reservoirs.

Although colistin-resistance have been detected in sewage/wastewaters in South America (Figure 1), the presence of *mcr* in these ecosystems in the continent is yet to be reported. Nevertheless, it is worthy to note that no *mcr*-1-carrying strain was detected among 7 COL-R enterobacterial isolates from sewage samples in Brazil [96]. Similarly, no strain among 35 enterobacterial isolates from sewage in Venezuela harboured *mcr*-1 [97].

### 3.3. Water (Fresh and Sea), Aquaculture and Aquatic-Based Foods

#### 3.3.1. Freshwater and Seawater Ecosystem

Water bodies (both surface and underground) are receptacles of anthropogenic wastes (from human and animal sources); thus they are reservoirs of ARGs [98]. Eighteen publications reported on plasmid-mediated colistin resistance in a total of 2107 isolates from fresh and seawater. These studies investigated *mcr* genes in a total of 1652 isolates and reported *mcr*-1 gene in 68 isolates (60 *E. coli*, 2 *Enterobacter*, 4 *Citrobacter* and 2 *Klebsiella*) and *mcr*-3 gene-type variants in 3 *E. coli* and 11 *Aeromonas* isolates, respectively. 

Dissemination of MGCB into the surface and underground waters have been reported in Europe. An *mcr*-1-carrying multidrug-resistant ST359 *E. coli* strain was detected among 74 ESBL-producing Enterobacteriaceae (1.35%) isolated from rivers and lakes in Switzerland [99], suggesting contamination of Swiss surface waters by COLROS capable of causing difficult-to-treat infections. In a study from Italy, an ST10 *E. coli* strain carrying *mcr*-1.2 together with β-lactam resistance genes (including ESBL gene) on IncX4 plasmid, was detected among 264 isolates (4.2%) from well/stream water [100], suggesting that underground waters in Italy are reservoirs of HiR zoonotic COL-R ExPEC [70,73]. The *mcr*-1-positive *E. coli* strain transferred COL resistance to a recipient organism at a very high rate of ~10^−2^ transconjugants per recipient, meaning it can rapidly spread the resistance genes to other organisms in the aquatic systems thereby posing public health risks. 

Similarly, 2 ESBL-producing ST10 *E. coli* strains carrying *mcr*-1 on diverse plasmids (IncFII, IncI1, IncFIB, Col156, IncX4 and ColRNAI) were detected among 83 coliform isolates (2.4%) from seawater at a public beach in Norway [101], suggesting that coastal seawater is a potential source for acquisition of HiR pandemic COL-R ExPEC clones [70,73]. Since ST10 is a zoonotic clone, its presence in seawater suggests it may have originated from humans bathing in the water, contamination from boat toilets, farm animals, fertilizers (manure) used in agriculture or migrating birds [101]. Presence of *mcr*-1-positive *E. coli* strains in coastal waters/public beaches is worrisome because individuals visiting these beaches for recreation and other purposes could get infected with organisms that potentially cause difficult-to-treat infections. Even worse was the fact that the *mcr*-1-positive *E. coli* strains harboured 8 other resistance genes in 5 different antimicrobial families (Table 3), further suggesting that seawater might be spreading a cocktail of multiple resistances, posing a significant threat to public health [102].

South American studies also reported the presence of MGCB in surface waters. Three multidrug-resistant *E. coli* isolates of ST10/phylogroup B1, ST46 and ST1638 carrying *mcr*-1 on ~33 kb IncX4 plasmid, were recovered from coastal public beach waters in Brazil [103], suggesting that Brazilian beaches are potential reservoirs of colistin-resistant HiR pandemic ExPEC clones thus posing serious public health risks, especially to those (such as residents, beach workers, tourists, and wildlife) directly exposed to this infectious threat from water exposure, contact with sand or through food consumption on the beach [70,73,103]. There were 15 extra resistance genes in 7 different antimicrobial families in the strains (Table 3), further suggesting that sea water is a reservoir for multiple resistance genes possibly emanating from anthropogenic/agricultural sources. However, the strains were susceptible to carbapenems. Of note, in another Brazilian study, no *mcr*-1-carrying strain was detected among 5 colistin-resistant enterobacterial isolates from lakes and rivers [96]. In another Brazilian study, no *mcr*-1- or *mcr*-2-carrying strain was detected among 40 Enterobacteriaceae isolated from urban lakes contaminated with human wastes [104]. However, these lakes contained ESBL-producing ST131 *E. coli*, which is a zoonotic pandemic HiR international ExPEC clone [70,73], thus highlighting the risk of poor environmental sanitation in Brazil.

MGCB has been detected in surface/underground waters in Asia. An *E. coli* strain carrying *mcr*-1 on IncI2 plasmid was isolated from water in Malaysia [105], suggesting that IncI2 may be a common plasmid spreading colistin-resistant resistance in Malaysian aquatic systems. From the same country, a retrospective study on 900 ST410 *E. coli* isolates collected in 2009 from pond water, detected one *mcr*-1-carrying strain (0.1%) [106], suggesting that MGCB has been circulating in Malaysia for the past 10 years. There were 13 resistance genes in 7 antimicrobial families in the *mcr*-1-positive *E. coli* strain (Table 3), further indicating that accumulated environmental surface waters are potential sinks for multiple resistance genes possibly originating from human/animal settings. In a Bangladeshi study, one *mcr*-3-carrying strain was detected among 12 carbapenem-resistant *E. coli* isolates (8.3%) from pond water [107], suggesting that surface water systems in Bangladesh are potential reservoirs of colistin-resistant as well as carbapenem-resistant organisms, thus posing a worrisome threat to public health. These COLROS possibly emanated from human/animal settings and they could spread from these ponds to other ecosystems by rainfall run-offs. Also, animals depending on these ponds for sustenance, could get infected with COLROS and consequently disseminate them to other ecological niches.

The presence of MGCB in irrigation water was reported in Asia. Twenty-two *mcr*-1-carrying *E. coli* strains were isolated from irrigation water in Lebanon [108], suggesting that anthropogenic/agricultural wastes have contaminated Lebanese irrigation system. This finding highlights the need for improved waste management in Lebanon, a country whose infrastructural facilities are being overstretched due to the prolonged Syrian war. Interestingly, the *mcr*-1-positive *E. coli* isolates survived in water for 45 days without losing *mcr*-1 implying that the gene persists in water matrix [108]. There were 5 genes encoding β-lactam resistance (including against carbapenem) and class 1 integrons in the strains (Table 3), indicating that diverse MGEs drive the spread of genes conferring resistance against last-resort antibiotics in the region. The presence of *mcr*-1-positive *E. coli* strains in the Lebanese irrigation system requires urgent attention because these superbugs could spread into the Mediterranean Basin contaminating plants and sea thereby spreading (by water current) to other parts of the world. In another Lebanese study, 32 *mcr*-1-carrying *E. coli* strains were isolated from domestic (drinking/well waters) and sewer-generated water samples collected from Syrian war refugee camps [109], suggesting that underground and drinking waters are a potential source/route for the acquisition of COL resistance. Possible sources of contamination of domestic water include sippage from sewers into underground water or human carriers. Class one integrons and β-lactam resistance genes (including carbapenem genes) were also present in the strains (Table 3), further indicating that diverse MGEs are involved in the spread of COL resistance in aquatic and other ecosystems in Lebanon. This report highlights the need for increased surveillance of domestic waters for MGCB, and improvement in the disposal of wastes as well as water treatment in the refugee camps. Survival of the strains in water for 30 days without the loss of *mcr*-1, further indicates persistence of the gene in water.

Several investigators from China reported the presence of *mcr* genes in the water ecosystems. In one study, the *mcr*-1 was detected on 33 kb IncX4 and 60 kb IncI2 plasmids, as well as on non-conjugative plasmids in *E. coli* isolates from seawater samples [85], further indicating that seawater is a route for spreading (by sea current, fishing or ships) COL resistance into aquatic ecosystems worldwide. It also further suggested that IncX4 and IncI2 plasmids are the major drivers of COL resistance in seawater ecosystems. In another study, 2 *Aeromonas caviae* strains carrying novel *mcr*-3 genes named *mcr*-3.13 and *mcr*-3.18 as well as *mcr*-3-like4 gene, were isolated from river/lakes/fountain water samples [110], suggesting that *mcr*-3 genes have disseminated into environmental waters in China. Two *mcr*-3.14- and *mcr*-3-like4-carrying *A. bivalvium* strains were also isolated from the same water samples. These strains contained 6 additional resistance genes in 4 antimicrobial families (Table 3) but exhibited susceptibility to some antibiotics, including polymyxin B, carbapenems and COL suggesting that *Aeromonas* species (common inhabitants of aquatic systems) are reservoirs of *mcr*-3 and multiple resistance genes thus posing a challenge to public health. 

In a further study, 2 *mcr*-1-carrying strains were detected among 6 colistin-resistant *E. coli* isolates (33.3%) from river samples [111], further suggesting the spread of COLROS in surface waters in China. There were transposon and class 1 integrons, including 6 resistance genes in 3 different antibiotic families in the organisms (Table 8), further indicating that diverse MGEs drive COL resistance in the Chinese environment. Similarly, 2 *mcr*-1-carrying strains were detected among 10 ESBL-producing *E. coli* isolates (20%) from well water collected from sites where chicken manure has been applied in the soil for a long time [62], suggesting that animal manure is a potential source of COLROS in underground water. Human carriers, sippage from septic tanks, as well as dejections from wildlife (such as reptiles and overflying birds) particularly if the well was not covered, might also be the sources of the *mcr*-1-positive *E. coli* strains. Nonetheless, it is worrisome that the *mcr*-1-positive *E. coli* strains were ESBL-producers and belonged to HiR zoonotic pandemic ExPEC ST10 and ST48 clones (both of ST10 complex) [70,73], thus posing health challenge to the public. These findings strongly suggest that in China, COLROS are circulating within the animal-human-environmental ecosystem because isolates in ST10 complex are the most common faecal *E. coli* strains detected in humans in China [62,112].

In a different study, 23 *mcr*-1-carrying *Enterobacteriaceae* (16 *E. coli*, 1 *Enterobacter cloacae* and 2 each of *Citrobacter freundii*, *Citrobacter braakii*, and *K. oxytoca*) were isolated from *mcr*-1-positive water samples [113], suggesting that although *E. coli* is the most commonly detected *mcr*-1-harbouring organism, transmission of this gene is not restricted to any species but in diverse organisms in aquatic environment thus posing public health risk. Seven other genes encoding β-lactams (including ESBL) resistance and PMQR were found in the *mcr*-1-positive *E. coli* isolates which were extensively diversified belonging to 6 different clones dominated by HiR pandemic clone ST10 [71,73], thus portending grave danger to public health. In another study, 25 *mcr* gene-positive isolates comprising 18 *mcr*-1-carrying isolates (17 *E. coli*, and 1 *Enterobacter cloacae*) and 7 *mcr*-3-carrying *Aeromonas* strains (*2 Aeromonas veronii* and 4 *A. hydrophila*) were detected among 1500 isolates (1.67%)from water samples collected from different points of a river [114], suggesting that COLROS are widely distributed in rivers in China. It further shows that diverse organisms are spreading COL resistance in aquatic systems, while *Aeromonas* seems to be a major reservoir of *mcr*-3. In addition, there was high concentration (2.0–2.7 log10 GC/mL) of *mcr*-1 and other resistance genes in the river and its surrounding environment, further indicating that contaminants in Chinese surface waters are the majority of anthropogenic/agricultural origins. 

Although there is a paucity of information from Africa, there are evidence that COLROS has disseminated into water ecosystem in the continent. Two tigecycline-resistant *E. coli* strains of ST23 and ST115 carrying *mcr*-1.5 on IncI2 plasmid and *mcr*-1.1 on IncHI2 A plasmid, respectively, were detected among 246 colistin-resistant isolates (0.8%) from seawater polluted with domestic, hospital, agricultural and industrial wastes [102,115], further indicating that anthropogenic/agricultural wastes (human and animal ecosystems in Algiers contain MGCB) are sources of pandemic HiR international COL-R ExPEC clones in seawaters [70,73]. This finding calls for serious concern because tigecycline is one of the last antibiotics used for managing deadly infections. There is need for rapid surveillance of COL and tigecycline resistance in African countries because in most of these nations, the use of antimicrobials in human/animal/environmental settings is not strictly controlled and sanitary facilities are lacking resulting in poor management of wastes which most often are disposed into the environment (eventually carried to surface waters by runoffs) and surface waters. Unfortunately coastal and rural dwellers in Africa use these waters for a variety of purposes (recreation, drinking, fishing, laundering, bathing) potentially exposing them to infection by superbugs. In another Algerian study, an *mcr*-1-carrying ST345 *E. coli* strain was isolated from 10 irrigation water samples [116], further indicating that water bodies in the country have been contaminated by diverse colistin-resistant *E. coli* clones originating from anthropogenic/agricultural wastes. 

#### 3.3.2. Aquaculture Environment and Aquatic-Based Foods

The heavy use of antimicrobials in fresh and saltwater aquaculture underline why aquacultural environments/aquatic based-foods is now increasingly recognized as major reservoirs and source of dissemination of antimicrobial resistance [12,28,47]. Eight publications assessed plasmid-mediated colistin resistance in a total of 838 isolates from aquaculture/aquatic-based foods. Seventy-two isolates (62 *E. coli*, 3 *Aeromonas* carried *mcr*-3 gene variants, 2 *Klebsiella pneumoniae* and 1 *Salmonella enterica* serovar Rissen) were reported to harbour *mcr* gene. One of the publications detected *mcr*-1 gene in seafood samples by direct sample testing. 

European studies reported MGCB in aquaculture/aquatic-based foods. A quinolone-resistant ST48/phylogroup A *E. coli* strain carrying *mcr*-1 on IncHI2, IncN and IncX3 plasmids was isolated from seafood (scampi) imported into Norway from Bangladesh [117], suggesting that seafood trade is a potential route for intercontinental dissemination of COLROS. There were 16 resistance factors in 11 different antimicrobial families, including heavy metals in the strain (Table 4), suggesting that contact or consumption of seafoods are sources for acquisition of multiple resistance genes thus posing a worrisome threat to public health. In a retrospective German study, 3 *Aeromonas* strains (1 *A. allosaccharophila*, 1 *A. jandei*, and 1 *A. hydrophila*) isolated from fishes in 2005–2008, were observed to carry novel chromosomally-encoded *mcr*-3.6, -3.8 and -3.9, respectively [118], suggesting that *Aeromonas* species potentially maintain diverse *mcr*-3 genes in aquatic environment. There were 8 other resistance genes in 2 different antibiotic families in the strains (Table 4), further suggesting that fishes are a potential reservoir of multi-resistance genes. Interestingly, the *mcr*-3 gene variants in the isolates were acquired and not part of the indigenous genomes suggesting that *mcr*-3 genes circulating in the aquaculture environment may not necessarily have originated from the aquatic environment. The *Aeromonas* strains/*mcr*-3 genes probably disseminated into the aquatic environment from human/animal settings since plasmid- and chromosomally-encoded *mcr*-3 genes have also been from humans/animals *Aeromonas* strains in the study region [110,119]. Detection of *mcr*-3 in a strain isolated in 2005 which is at least 10 years older than all the *mcr*-3-positive isolates from China suggests that *mcr*-3 gene group has been present for at least 12 years in Europe [118].

Recently in Galicia Spain, an *mcr*-1-carrying ST469 *Salmonella enterica* Serovar Risen strain was detected among 19 *Salmonella* isolates (0.3%) from 5907 mussels collected from processing facilities [120], suggesting that these bivalve molluscs were captured in water bodies contaminated by anthropogenic/agricultural wastes such as sewage discharges/outfall, combined sewer overflows, rainwater, aquaculture and wildlife discharges since ST469 *Salmonella* Rissen clone have been reported in livestock [121]. There were 9 other resistance genes in 6 different antimicrobial families in the strain (Table 4), suggesting that MDR *Salmonella* could be acquired from mussels thus posing a worrisome threat to the handlers and consumers of seafood. This finding of *mcr*-positive *Salmonella* strain in Galician mussels calls for concern because Galicia is the third-largest producer of cultured mussel worldwide and is considered the leading supplier of mussels to the European market [120]. It is also worthy to note that in Portugal, none of *mcr*-1 to *mcr*-5 was detected in isolates from aquaculture environment [122]. 

In the recent past, intensive aquaculture in China was characterized by heavy use of antimicrobials, including COL [12,47]. Some Chinese investigators reported the presence of *mcr* genes/MGCB in the aquaculture environment/aquatic-based foods. The *mcr*-1 was detected in 9 of 63 (14%) and 2 of 12 (17%) seafood samples sourced locally and from overseas, respectively [85], further suggesting that local and international trade of aquatic-based foods are potential routes for the dissemination of MGCB. Multidrug-resistant *E. coli*, *K. pneumoniae* and *A. veronii* strains susceptible to some antibiotics, including meropenem and tigecycline were isolated from the *mcr*-1-positive samples, further indicating that diverse multidrug resistance organisms could be acquired from sea-based foods. In another Chinese study, 7 strains carrying *mcr*-1 on different plasmids (IncP, IncX4 and IncI2) and on the chromosome were detected among 190 *E. coli* isolates (3.7%) from cultured grass carp fish [123], further suggesting that COL resistance is circulating by horizontal and vertical transfer among isolates from aquatic ecosystems. There was diversity among the isolates (ST7508, ST2040, ST156 and ST48), suggesting that promiscuous plasmid types resulted in a diverse range of *mcr*-1-carrying clones in aquaculture environment. In addition, conjugation was positive at a very high frequency, and there were 19 other resistance genes in 9 different antimicrobial families plus a composite transposon in the strains, thus suggesting that the organisms could rapidly transfer COL and multiple resistances, thus posing a serious challenge to public health. 

Integrated livestock-fish farms have also been reported to be reservoirs of COL resistance in China. Recently, 54 *E. coli* and 2 *K. pneumoniae* strains carrying *mcr*-1.1 on different plasmids (IncHI2, IncI2, IncX4, IncP, and Incp0111) and 4 *mcr*-3-carrying *Aeromonas* strains, were detected among 143 colistin-resistant isolates from samples collected from duck-fish integrated fishery [28]. There was IS*ApI1* upstream of *mcr*-1 in the *mcr*-1- positive *E. coli* isolates which were extensively diverse belonging to 5 ST with the zoonotic ExPEC clone ST93 being the predominant [71,73] (Table 4). This further indicates that diverse MGEs transfer COL resistance in diverse clones of *E. coli* in livestock/aquatic ecosystems in China. It also further indicates that *Aeromonas* is a potential reservoir of *mcr*-3 in livestock/aquatic ecosystems. Remarkable, 2 ST156/phylogroup B1/cluster C2 *mcr*-1-positive *E. coli* strains from a duck and a fish, had zero SNPs between them indicating that integrated farming is a route for transferring *mcr* genes between livestock and aquaculture [28]. Additionally, the *mcr*-1-positive *E. coli* isolates from the integrated farms were genetically related to those from humans in the farm region, thus suggesting that MGCB transfer between animals and humans via the aquatic food chain [28]. There is a dearth of information on plasmid-mediated colistin resistance in aquaculture ecosystem in other Asian countries. However, it is worth noting that no *mcr*-1- or *mcr*-3-carrying strain was detected among 6 colistin-resistant *Klebsiella* isolates from fish in India [124]. Contaminated human carriers, improper disposal of human and animal sewage to the aquatic environment, feeding of fish with contaminated feed or animal manure especially in integrated farms, dejections of wild animals, and/or the emergence of *mcr* genes from aquatic organisms, are possible sources of MGCB in aquatic/aquaculture environment [56,123]. Therefore, to curb the spread of COL resistance through the trade of aquatic products, increased surveillance of aquaculture is warranted [123].

## 4. Soil/Manure Ecosystem

There is a growing interest about the role of animal manure and other resistance reservoirs (such as sewages/wastewaters/sludges, aquaculture and wildlife) in the transmission of resistance genes to the soil which constitutes a source for dissemination of these genes to botanical, aquatic and wildlife ecosystems [22,23,24,25]. Nine publications investigated on plasmid-mediated colistin resistance in a total of 276 reported isolates from soil/manure/slurry/sediment. Thirteen isolates (14 *E. coli* and 2 *Enterobacter*) were reported to harbour *mcr*-1 gene. Three of the study quantified *mcr*-1 gene in soil/manure samples. 

Studies from South America reported the presence of *mcr* gene in soil. In a Brazilian study, *mcr*-1 was detected in soils from vegetable production areas that received non-composted poultry litter as organic fertilizer as well as in native vegetation areas without livestock manure [125], suggesting that the COL resistance in the soil does not necessarily occur only when livestock manure has been used. Anthropogenic wastes (especially from industries, hospitals, and laboratories) that may contain antibiotics, aquaculture, sewages/wastewaters and wildlife discharges, are putative sources of COL selection pressure in the soil where livestock manure has never been applied. Although not surprising, the *mcr*-1 was more abundant in the fertilized vegetable production area than in the native vegetation area, suggesting that non-composted poultry litter is a potential source of *mcr*-1/MGCB in the soil. 

European studies also reported that manure/soil is reservoirs of *mcr* genes/MGCB. Two *E. coli* strains of ST5281 and ST1011 carrying *mcr*-1 on diverse plasmids (IncI1, IncFII, IncFIB, IncX1 and IncQ1) were isolated from manure collected close to pig farms in Germany [65], suggesting that livestock manure is a reservoir of promiscuous plasmids resulting in diverse range of *mcr*-1-positive *E. coli* clones in manure/soil environment. These organisms also harboured 17 resistance genes in 5 different antimicrobial families (Table 8) indicating they are multidrug-resistant thus posing a serious risk to public health, especially to the handlers of the manure, crop farmers, and consumers of plant products from farms fertilized with these manures (Table 8). Interestingly, there was close-relatedness between the ST1011 isolate to another *E. coli* isolate from a stable fly (*Musca domestica*) collected from a close distance to another pig farm in the same country, thus suggesting that flies are potential vectors of MGCB transferring these organisms from livestock farms to other ecological niches. Since these garbage flies usually feed on and breed in animal manure [57], there is a need for increased environmental sanitation especially regarding the disposal of animal manure. In an Estonian study, 3 *E. coli* strains carried *mcr*-1 on IncX4 plasmid were detected among 141 ESBL-producing isolates (2.6%) from pig slurry originating from a farm [126], further indicating that animal farm wastes are potential sources for the spread of COL resistance to the soil and other ecosystems. There were IS26 upstream of *mcr*-1 and 3 genes (including ESBL and pAmpC gene) in the *mcr*-1-positive *E. coli* strains, suggesting that different MGEs (insertion sequences and plasmids) drive COL resistance in manure/soil environment.

In Asia, investigators documented *mcr* genes/MGCB in the soil/manure ecosystem. In a recent Lebanese study, 3 *mcr*-1-carrying *E. coli* strains belonging to different clones were isolated from 41 poultry litter/faecal samples [116], further suggesting that animal (poultry) manure is a source of dissemination of COL resistance into the environment. In a study from China, 6 strains carrying *mcr*-1 on IncH12 plasmid and chromosome were detected among 10 colistin-resistant *E. coli* isolates (60%) from farming soil where there is intensive livestock farming [127], suggesting that IncH12 is a major driver of COL resistance in isolates from soil/manure ecosystems in the country. There was chromosomal integration of *mcr*-1 in some of the strains implying vertical transmission of *mcr*-1in *E. coli* strains from soil/manure. Thirty-five other resistance genes in 10 different antimicrobial families were harboured by the *mcr*-1-positive *E. coli* strains (Table 5), indicating that soil especially where livestock farming is practiced, is a huge reservoir of multiple resistance genes thus posing a worrisome threat to public health especially to individuals directly in contact with contaminated soil. In another Chinese study, high concentration (1~10^5^) of *mcr*-1 was detected in faecal/soil samples from a chicken farm [128], further suggesting that soil constitute a huge reservoir for COLROS/*mcr* genes. A similar study detected a higher concentration (1.87 × 10^7^–1.82 × 10^9^ copies/g dry weight) of *mcr*-1 in 16 of 51 manure samples (1.4%) from farms in China [129], suggesting that *mcr*-1 is widely distributed in animal manures in the country. Encouragingly, there was significant (>90) reduction in the quantity of *mcr*-1 after 15 days of composting suggesting that by composting, *mcr*-1 can efficiently be eliminated from livestock manure thereby preventing its spread to the environment [129]. Some other Chinese investigators showed that banning the use of COL resulted in a reduced concentration of the antibiotic and *mcr*-1 in feed and fresh manure [130], thus suggesting that COL in manure exerts direct selection pressure for the accumulation of *mcr*-1 and that banning non-therapeutic COL use may curb the spread of MGCB/*mcr* genes. The same Chinese group also showed that anaerobic digestion of animal manure reduces the number of MGCB better than natural drying. Evidence that soil/animal manure are reservoirs for MGCB has also emerged from Africa. Very recent, 5 *mcr*-1-carrying and 2 *mcr*-3 carrying *E. coli* strains were detected among 28 isolates (17.9%) from soil/animal manure collected from agricultural sites/farms in Algeria [131], further indicating that animal manure is a source of spread of different *mcr* gene-types in Algeria. The strains were multidrug resistance phenotype and they were diverse belonging to HiR pandemic zoonotic ExPEC clones (ST405, ST10, and ST155) [70,73], thus posing a threat to public health, especially to the farmers and when water run-offs transport the pathogens to other ecosystems. This highlights the need for composting/anaerobic digestion of animal manure before disposal into the environment. There was ESBL gene in the ST405 isolates, a known clone for globally dissemination ESBL genes [55]. This clone was associated with diseases in humans and wildlife in Algeria [55,132], suggesting it has disseminated into diverse ecosystems in the country. Unfortunately, conjugation was positive in some of the strains suggesting that the strains could transfer COL resistance to organisms in various ecosystems.

## 5. Botanical Ecosystem

The importance of soil as a source of antimicrobial-resistant organisms contaminating plants (vegetables and fruits) is well recognized [22,23,24,25]. Plant-based foods are often consumed raw or undercooked hence potential route for dissemination of antimicrobial-resistant organisms as well as emerging pathogens thus posing a grave danger to public health. Eight publications investigated on plasmid-mediated colistin resistance in a total of 746 isolates from plant (fruits and vegetables) samples. One of the studies probed *mcr*-1 gene directly in the samples prior to isolation. Thirty-one isolates (28 *E. coli*, 2 *Raulotella ornitholytica* and 1 *K. pneumoniae*) were reported to harbour *mcr*-1 among the isolates tested.

Studies from European countries reported the presence of MGCB in vegetables. Two *mcr*-1-carrying *E. coli* strains of ST167 (ExPEC clone) and ST4683, were detected among 60 ESBL-producing Enterobacteriaceae (3.3%) isolated from vegetables imported into Switzerland from different countries [99], suggesting that international plant-based foods trade is a potential route for global dissemination of diverse virulent clones of *mcr*-1-positive *E. coli* [71,73], thus posing serious threat to public health. In a Portuguese study, one pathogenic *mcr*-1-carrying ST1716 *E. coli* strain was detected among 138 isolates (0.7%) from conventional fresh vegetables [133], further indicating that these vegetables are a source of COLROS in Portugal. This poses serious risks to the health of handlers and consumers especially those that consume vegetables raw or undercooked. Possible sources of the *mcr*-1-positive *E. coli* include soil, irrigation water or wildlife ejections. There were class 2 integrons, 6 prophage regions, and 8 other resistance genes in 6 different families of antimicrobials in the strains suggesting that the genes were horizontally acquired by transduction (since bacteriophages often attack organisms colonizing plants) and/or other means. It also suggested that integrons are common drivers of COL and multiple resistance in botanical ecosystems. The occurrence of MGCB in vegetables has also been reported in Asia. In a study from China, *mcr*-1-carrying multi-drug resistance *E. coli* strains were detected in 18 out of 271 vegetable samples (7%) [85], further suggesting that vegetables are reservoirs of MGCB in the country. These *mcr*-1-positive *E. coli* strains possessed similar traits, as described above. In another study from China, 9 strains (7 *E. coli* and 2 *Raoultella ornithinolytica*) carrying *mcr*-1 on chromosome and diverse plasmids (IncX4, IncI2 and IncHI2/ST3) of varying sizes, were detected among 270 ESBL-producing isolates (3.3%) from vegetables [134], suggesting that these plasmids are drivers of COL resistance in diverse organisms from plants and that *mcr*-1 could persist in plants ecosystems having integrated in chromosome of these organisms. There was extensive diversity among the *mcr*-1-positive *E. coli* isolates that were in 6 STs with some of them belonging to the ExPEC clones (ST156, ST69 and ST48) [70,73]; these isolates also possessed IS*ApI1* upstream of *mcr*-1 and harboured 5 other resistance genes in 4 different antimicrobial families indicating that diverse MGEs drive the spread of COL resistance in botanical environment just like in other ecosystems and that handlers/consumers of these vegetables could be exposed to virulent COLROS capable of causing difficult-to-treat diseases. 

In a recent Chinese study, 25 *mcr*-1-carrying enterobacterial strains (24 *E. coli* and 1 *Enterobacter*) were isolated from 19 vegetable samples [135], further supporting that diverse species of COLROS colonize vegetables. There was no clonal restriction in the acquisition of *mcr*-1 among the *mcr*-1-positive *E. coli* isolates and they were extensively diverse belonging to 16 STs dominated by ST744 and ST224. Transposons and insertion sequences, as well as 14 resistance genes in 7 antimicrobial families, were present in the strains (Table 6). These findings suggest that diverse MGEs drive COL resistance in various *E. coli* clones in China. In another new Chinese study, 2 carbapenem/fosfomycin-resistant *E. coli* isolates carrying *mcr*-1 on ~33 kb IncX4 plasmid (in ST156 isolate) and ~60 kb IncI2 plasmid (in ST2847 isolate), were isolated from vegetables [136], suggesting that resistance against last resort antibiotics could be acquired by having contact with/consuming vegetables, thus posing serious risks to public health. However, the strains exhibited susceptibility to amikacin and tigecycline similar to *E. coli* strains from humans in China that also harboured both carbapenem and COL resistance genes [137], suggesting that MGCB are circulating from the human setting (through sewages/wastewaters and irrigation waters) to plant ecosystem in the country.

Fruits were reported as the source for dissemination of plasmid-mediated colistin resistance in China. An ST189 *E. coli* and an ST442 *K. pneumoniae* strain carrying *mcr*-1 on IncFIA and IncHI1 plasmids, respectively, were detected among isolates from marketed fruits [138], suggesting that fruits are potential reservoirs of diverse *COLROS thus fruit* trade is a potential route for dissemination of these organisms. There were 18 resistance genes in 9 different antimicrobial families in the strains (Table 6), suggesting they are MDR thus posing a troubling threat to public health. Worse of it is that the ST442 *K. pneumoniae* is a progenitor of the widely spread multidrug-R ST258 *K. pneumoniae* strain while the *E. coli* contained *ast*A gene encoding heat-stable enterotoxin-1 associated with diarrhoea in humans [138]. It is worth mentioning that no *mcr*-1 or *mcr*-3-carrying strain was detected among 17 colistin-resistant *Klebsiella* isolates from vegetables/fruits in India [124]. However, chromosomal *mgr*B alterations was detected in some of the strains, suggesting that diverse mechanisms mediate COL resistance in environmental isolates [75,76,77]. 

There is no report on MGCB colonizing plants in the North and South America and Africa. Therefore, surveillance of plasmid-mediated colistin resistance in botanical ecosystem is warranted in these continents. Nevertheless, it is worth noting that no *mcr*-1 or *mcr*-2-positive strain was detected among 240 shigatoxin-producing *E. coli* isolates from vegetables/fruits in the US [139], and no *mcr*-1-carrying strain was detected among COL-resistant enterobacterial isolates from vegetables in Brazil [96]. 

It is evident that vegetables and fruits are reservoirs and source of spread of MGCB; therefore, proper disposal of animal excrement before use as fertilizer and improvement of irrigation water need to be taken [134]. 

## 6. Wildlife (Birds, Mammals, Reptiles, and Flies)

Because antimicrobials are not used in the wildlife, the presence of any acquired ARG in the absence of selection pressure often indicates transfer from other ecosystems [50,51,52,53,54,55]. Seventeen publications investigated on plasmid-mediated colistin resistance in a total of 1073 isolates from wildlife (birds, flies and mammals). Four of the studies probed *mcr* gene directly in the samples. A total of 113 isolates (67 *E. coli*, 4 *Pseudomonas*, 7 *Enterobacter* and 27 *K. pneumoniae*) harboured *mcr* gene. Fifty-six *E. coli*, 6 *Enterobacter* and 2 *K. pneumoniae* isolates harboured *mcr*-3 gene, respectively. The *mcr*-1 gene was observed in one *Enterobacter*, 56 *E. coli*, 4 *Pseudomonas* and 17 *K. pneumoniae*, respectively while *mcr*-8 gene was detected in 17 *K. pneumoniae*. Two flies were reported to carry the *mcr*-2 gene.

### 6.1. Wild Birds

Studies from Europe reported wild birds as potential reservoirs of *mcr*-1-positive *E. coli*. A *mcr*-1-carrying ESBL-producing strain was detected among 177 *E. coli* isolates (0.6%) from European herring gulls (*Larus argentatus*) in Lithuania [140], suggesting that these migratory birds can potentially spread genes conferring resistance against last resort antibiotics from Europe to other places, especially south where the birds move to during winter [140]. The birds could potentially spread COL resistance to water ecosystems since they migrate through the Baltic sea. In a Spanish study, an *mcr*-1-carrying pAmpC-producing ST162 *E. coli* strain was isolated from a black vulture [141], suggesting that scavenging animals could acquire COLROS from habitats contaminated by anthropogenic/animal wastes and then disseminate them to other locations. Scavenging on carrions, slaughterhouse wastes and flies potentially exposes vultures to colonization by antimicrobial-resistant organisms. The investigators detected HiR pandemic international ST131 ExPEC strains that harboured genes encoding ESBL, pAmpC and carbapenemases, indicating wide distribution of multidrug-resistant genes in the Spanish environment [141]. Therefore, wild birds can disseminate these genes encoding resistance to last-resort antibiotics to diverse ecological niches posing a serious threat to public health. It is also worth noting that no strain among 19 isolates from urban birds (yellow-legged gulls, pigeons and chickens) in France harboured *mcr*-1 to *mcr*-5 [142].

Migratory wild aquatic birds in South America were reported to harbour *mcr*-1-carrying *E. coli*. An ESBL-producing ST10/phylogroup A *E. coli* strain carrying *mcr*-1 on 33 kb IncX4 plasmid was isolated from a Magellanic penguin (*Spheniscus magellanicus*) suffering from pododermatitis [143]. In the strain, diverse plasmids carried 5 other resistance genes in 5 different antimicrobial families (Table 7), thus suggesting that multidrug-resistant HiR zoonotic ExPEC clones have acquired resistances from various sources and have spread widely in the environment [71,73]. Similarly, 5 ESBL-producing *E. coli* strains (of ST744 and ST1010) carrying *mcr*-1 on ~57 kb IncI2 plasmid were isolated from Kelp gulls (*Larus dominicanus*) in Argentina [144], suggesting that wild migratory birds could potentially disseminate diverse *mcr*-1-carrying *E. coli* clones to other parts of the globe since these gulls fly across continents [144]. There was IS*ApI1* upstream of *mcr*-1 and conjugation was at a very high frequency of ~2 × 10^−6^, further suggesting that diverse MGEs facilitate rapid spread of COL resistance in wild aquatic habitats. 

Asian studies also reported wild migratory birds as potential carriers of *mcr*-1-carrying *E. coli*. An ESBL-producing ST354 *E. coli* carrying *mcr*-1 on 63 kb IncI2 plasmid was isolated from a long-range wild migratory waterbird Eurasian coot (*Fulica atra*) in Pakistan [64,145], further suggesting that IncI2 is one of the major plasmids driving the spread of *mcr*-1 in wildlife ecosystems. Since Eurasian coots migrate from Europe to Asia, they could potentially disseminate MGCB into water bodies and other ecosystems to and from these continents, thus posing risks to the health of aquatic animals and the public [64,145]. In a Chinese study, one *mcr*-1-carrying multidrug-resistant strain was detected among 6 colistin-resistant *E. coli* isolates (16.7%) from faecal samples collected from egret habitat [111], further supporting that aquatic wild birds disseminate COL resistance to various ecosystems. It is worth noting that another Chinese study focusing on ESBL-producing *E. coli*, observed 20% phenotypic COL resistance among strains from wild birds [146]. 

Wild birds are potential reservoirs of COLROS. These birds might have picked the MGCB by contact with contaminated water, or consumption of food material or drinking water contaminated with anthropogenic/agricultural wastes or from other ecosystems.

### 6.2. Wild Mammals

So far, only a study reported MGCB in a wild mammalian species. Bachiri et al. [55] isolated an *mcr*-1-carrying ST405/phylogroup D *E. coli* strain from a Barbary macaques monkeys (*Macaca sylvanus*) in Algeria, suggesting that HiR pandemic zoonotic ExPEC clone is circulating COL resistance in the wild. There were genes encoding ESBL and PMQR in the strain, further suggesting that wildlife potentially disseminate genes conferring resistance to last-resort antibiotics [50,51,52,53]; it also supports that ST405 *E. coli* clone disseminates ESBL worldwide [55]. ExPEC ST405 was associated with infertility and urinary tract infection in humans in Algeria [132,147] and it has been found in livestock and environment in the country [131], thus suggesting that this zoonotic pandemic clone is circulating in human/animal/environment ecosystems in Algiers. Direct contact with human/animal dejections, eating vegetables/fruits or drinking contaminated water is possible routes by which the monkey acquired the ST405 ExPEC clone. Conjugation was negative suggesting that *mcr*-1 was located on the chromosome in the strain, hence can be maintained/persist in the wild. It is worth noting that no *mcr*-1-carrying strain was detected among 54 *E. coli* isolates from rodents in China [68]. Also, no *mcr*-1-harbouring strain was detected among 269 faecal *E. coli* isolates from wild mammals/birds in the UK [148].

### 6.3. Flies

The role of flies as vectors for transmission of antimicrobial-resistant organisms is well recognized [57,58]. Their synanthropic nature linking human, animal and environmental ecosystems allows them to be colonized by diverse organisms and to deposit these organisms in various ecological niches [57].

Flies were reported as carriers of MGCB in Europe. An ST1011 *E. coli* carrying *mcr*-1 on IncX4 plasmid was isolated from a barn stable fly (*Musca domestica*) captured from a 50–150 m distance to a pig farm [65], suggesting that flies potentially transmit COLROS from one spot to another even to far distances. It also suggested that IncX4 is spreading *mcr* to and from diverse ecosystems. The strain harboured 9 other resistance genes in 5 different antimicrobial families on diverse plasmids (Table 8), suggesting that flies transmit multiple resistance genes together, thus posing serious public health risk. It is worth mentioning that in a retrospective German study, none among 24 ESBL-producing *E. coli* isolates from 21 flies were observed to harbour *mcr*-1-like to *mcr*-8-like genes [74].

Asian studies also reported flies as potential reservoirs of MGCB. Four enterobacterial strains carrying *mcr*-1 on IncI2 plasmid with transposons (IS*Ecp1* and IS*Sen6*), were isolated from flies in China [86], further supporting that diverse MGEs are driving COL resistance in wildlife, including in flies. These strains have the capacity to rapidly transfer *mcr*-1 to other organisms since having transferred the gene to a recipient organism at very high frequencies of 9.0 × 10^−10^ to 5.0 × 10^−3^ cells per donor cell. Interesting, direct sample testing detected the presence of *mcr*-1 and carbapenem genes in various environmental niches (wild bird nests, dog kennel and poultry farms) some of which were regarded negative for the genes by isolation method, thus indicating that environmental niches constitute huge reservoirs of genes conferring resistance to last-resort antibiotics (phantom resistomes) which might be underestimated [86]. Therefore, direct sample testing followed by isolation could be the best approach for surveillance of resistance to last-resort antibiotics in environmental niches. In another Chinese study, an *Enterobacter* *cloacae* carrying *mcr*-1 on IncX4 plasmid and a *Raulotella planticola* harbouring *mcr*-1.3 were isolated from flies captured in poultry farms [149], suggesting that IncX4 is a common plasmid spreading *mcr* in the wild and that flies potentially transfer diverse organisms, including uncommon COLROS, from livestock farms to other ecosystems and *vice versa*. The feeding habit of flies allows them to be colonized by diverse organisms present in various ecological niches [57]. There was IS*Apl1* flanking *mcr*-1.3 in the *R. planticola*, further supporting that diverse MGEs are involved in the acquisition/spread of genes conferring resistance against last resort antibiotics in the environment. 

In a recent Chinese study, *mcr*-1, *mcr*-2 and *mcr*-3 was detected in 109 flies (86 *M. domestica* and 23 *P. terraenovae*) captured in public places and near dumpsites [150], suggesting that flies potentially harbour organisms carrying *mcr* gene-types possibly originating from other ecosystems and that flies transfer these to human habitations posing serious public health risk such as in food, water and wound contamination. Subsequent culture of the *mcr*-positive samples yielded only *mcr*-1-containing strains (4 *E. coli*, 2 *P. stuartii*, 2 *P. alcalifaciens*, and one *E. cloacae*), further suggesting that by isolation method, some COLROS might be missed resulting in underestimation of the magnitude of COL resistance in an ecological niche. Similarly, in a Bangladeshi study, 4 *mcr*-3-carrying strains were detected among 40 carbapenem-resistant *E. coli* isolates (10%) from 60 flies (*M. domestica*) [107], further suggesting that *mcr*-3 has spread in diverse ecosystems in Bangladesh.

In a study from Thailand, *mcr*-1 and *mcr*-3 were detected in 16 flies collected from urban areas and livestock farms [151], further indicating that flies are colonized by COLROS in diverse ecological niches in the country. However, only *mcr*-3-carrying strains (11 *E. coli*, 2 *Enterobacter aerogenes*, 4 *Enterobacter cloacae* and 2 *K. pneumoniae*) some of which also contained ESBL and pAmpC genes were isolated from the samples. This further supports that direct sample testing before isolation is a better approach for antimicrobial resistance surveillance. In another Thai study, 48 enterobacteria (17 ST43 *K. pneumoniae* and 31 *E. coli*) carrying *mcr*-1 on IncX4 plasmid were isolated from 300 blowflies (*Chrysomya megacephala*) (16%) collected from a local market in an urban community, a rural area and a city suburb [152], suggesting that MGCB is widely distributed in human environment in Thailand and that IncX4 plasmid is a major driver of *mcr*-1 in strains from flies. Unfortunately, *mcr*-8 was also present in all the *K. pneumoniae* strains, suggesting that flies potentially transmit virulent *K. pneumoniae* clones. The ST43 *K. pneumoniae* has been associated with abdominal infections, bacteraemia and intensive care unit infections [152]. Equally worrisome is that the *mcr*-1-positive *E. coli* strains were extensively diversified with HiR ExPEC clone ST10 dominating among 12 STs (Table 7). More of 20 resistance genes in 9 different antimicrobial families were found in the ST10 isolates, further indicating that flies can potentially spread virulent COLROS carrying multiple resistance genes, thus portending threat to public health. Furthermore, the *mcr*-1-positive *E. coli* strains transferred COL resistance to a recipient organism at a higher frequency than by *K. pneumoniae* strains, suggesting that *E. coli* transfers *mcr*-1 more rapidly than *Klebsiella*. Moreover, of the plasmids, the IncX4 plasmid was transferred at a higher mean frequency than the other plasmids (IncHI1A, IncHI1B, and IncHI1A-IncHI1B) further supporting that IncX4 is a major driver of *mcr*-1. This plasmid was further proven to potentially reduce the virulence of MGCB by injecting an ~1 × 10^5^ CFU of ST34 *K. pneumoniae* strains into *Galleria mellonella* larvae and observing significantly lowered mortality. It is worth mentioning that none of the studies on COL resistance among isolates from reptiles such as in turtles in Brazil, [96], reptiles in the US [139], and snake in Taiwan [153], detected any *mcr*-carrying strain. This warrants further surveillance since reptiles make contact with aquatic and terrestrial ecosystems.

## 7. Concluding Remarks

This review showed that diversity of MGCB such as *E. coli*, *Enterobacter*, *Klebsiella*, *Proteus*, *Salmonella*, *Citrobacter*, *Pseudomonas*, *Acinetobacter*, *Kluyvera*, *Aeromonas*, *Providencia*, and *Raulotella* have disseminated into environmental reservoirs, including contact surfaces in hospitals, public transportation routes and livestock farms, soil/manure/sludge, plants (vegetables and fruits), aquatic (aquaculture, seawater, ground and surface waters, sewage and wastewaters), and wildlife. These reservoirs are potential sources for further dissemination of *mcr* genes. Anthropogenic activities such as defecation in open environment/water, bathing/swimming in water bodies, improper disposal of the slaughterhouse, home, hospital and laboratory wastes, inappropriate use of antimicrobial agents in humans, animals/aquaculture and plants, are the major causes of dissemination of *mcr* genes into the environment.

Environmental isolates harbour *mcr* genes together with many virulence and resistance genes, including those conferring resistance against last resort antimicrobials. These organisms are superbugs capable of causing untreatable infections with pandemic potential. If unchecked, these organisms may diffuse into the human and animal ecosystems and present a challenge to control AMR [154]. Some environmental isolates have acquired megaplasmid with numerous ARGs (some harbour ≥10 genes). A further transmission of MGCB harbouring megaplasmid from the environment to human and animal ecosystems may result in the actualization of the O’Neill’s projection of 10 million AMR infection-associated deaths per 2050 [21]. Carbapenems and tigecycline, as well as some other commonly used antimicrobial agents, seem to be effective against most isolates in this review. The implementation of antibiotic stewardship programmes should preserve the efficacy of these last resort agents which could be used in treating cases associated with MGCB.

Drivers of plasmid-mediated colistin resistance facilitating horizontal/lateral transfer of *mcr* genes in the environment are diverse genetic elements, including conjugative plasmids of different replicons and incompatibility, class 1–3 integrons, transposons, complete, and truncated insertion sequences. IncHI2, IncI and IncX4 plasmids seem to be the predominant plasmid types harboured by isolates from different environmental reservoirs worldwide. The *mcr*-carrying environmental strains have the potential to spread worldwide since they transferred *mcr* gene-bearing plasmids to recipient strains at a very high frequency [15]. Nevertheless, *mcr* gene has integrated into chromosomal DNA and/or non-conjugative plasmids of environmental strains enabling the transfer of these genes to their progenies by vertical transmission thus ensuring the persistence of *mcr* genes among clonal lineages [155]. Transmission of *mcr* gene among environmental strains is clonally unrestricted and diverse highly virulent zoonotic pandemic and epidemic clones of *E. coli* and *Klebsiella pneumoniae* are circulating in environmental ecosystems worldwide.

Colonization of wildlife by MGCB implies that COL plasmid is maintained in bacterial populations regardless of antimicrobial selective pressure [114]. Since some *mcr*-1-linked plasmids like IncI2, IncX4 and IncHI2 plasmids (which are predominant in environmental isolates) could persist and increase the fitness of their host cells, MGCB in an environment such as wildlife without antibiotic pressure, may have an advantage [41,156,157].

Global production and trade of fresh plant produce and aquatic-based foods constitute potential routes of dissemination of MGCB. Integrated farms are sources of transfer of *mcr* genes into aquaculture which in itself have been associated with a high rate of human colonization by MGCB [28,158]. However, since livestock-fish integrated farming are considered economical and efficient farming modes in most developing countries, there is a compelling need for assessment and supervision of antimicrobial use and spread of ARGs within the aquaculture industry [28].

As demonstrated, banning of the use of COL other than therapy in livestock will curb the spread of MGCB from animal to human and environmental ecosystems. Commendably, some countries in the European Union as well as others like China, Brazil and Argentina, has taken the lead in enforcing the ban on the non-therapeutic use of COL [158].

Some of the isolates considered negative in various studies might harbour *mcr* gene types other than those assayed. This warrants an urgent need for affordable methods that can detect all the currently known *mcr* gene-types (*mcr*-1 to *mcr*-9, and the ones that are yet to emerge) for rapid and adequate surveillance of plasmid-mediated colistin resistance. Subjecting *mcr*-carrying isolates to high throughput analysis such as next-generation sequencing would help to provide information about the genetic context of the gene, elucidation of *mcr* genes that could be missed by other molecular techniques as well as the phylogenetic relationship of the isolates [114]. This information is crucial for understanding the epidemiology of COLROS and devising effective control strategies to reduce public health risks.

Since COL determinant emerging from any part of the globe can rapidly spread worldwide by international travel (even short distance travel) and food trade, there is a need for increased surveillance of *mcr* genes in environmental reservoirs, especially in Africa where the use of COL is largely uncontrolled, and sanitation is poor, and South America where public and environmental sanitation is also considered suboptimal [152]. Indeed, it is evident that by horizontal/lateral and vertical transfer, *mcr* genes (*mcr*-1, *mcr*-2, *mcr*-3, *mcr*-5, *mcr*-7, and *mcr*-8) have spread widely into diverse environmental niches (Figure 1). Thus, these ecosystems constitute underestimated vast reservoirs (‘phantom resistome’) of these *mcr* genes. This further underlines the need for One Health approach.

**Table 2:** Studies reporting plasmid-mediated colistin resistance in sewages/wastewaters.

Country	Source of Isolate	Date of Isolation (*mcr* Gene Assayed)	Number of Isolates Tested for *mcr*	Identified Gene/Variant (Number of Organism)	Sequence Type and/or Phylogroup (Virulence Genes)	Plasmid (Associated Insertion Sequence)	Additional Resistance Traits	Key Points/Conclusion	Reference
China	Hospital, wastewater	2013–2016	-	*mcr*-1 (8 *E. coli*)	-	-	-	- *mcr*-1originated from agriculture and spread to human and environmental ecosystems by transposon, IS*Apl1* and plasmids	[2]
China	Hospital sewage	2017(*mcr*-1)	1	*mcr*-1.1 and *mcr*-3.5 (1 *E. coli*)	ST410	IncHI2 and IncN	*aac(3)-IVa*, *aac(3)-IId*, *aac(6′)-Ib-cr*, *aadA22*, *aadA16*, *aph(3′)-Ia*, *aph(3′)-Ib*, *aph(6′)-Id*, *aph(4)-Ia*, *rmtB*, *strA*, *bla*_CTX-M-65_, *bla*_CMY-2_, *bla*_NDM-5_, *bla*_TEM-1B_, *fosA*, *lnu(F)*, *mphA*, *floR*, *arr-3*, *aac(6′)-Ib-cr*, *oqxA*, *oqxB*, *sul1*, *sul2*, *tet(A)*, *tet(M)*, *dfrA14* and *dfrA27*.	- Environmental strains can transfer *mcr*-1, carbapenem and high-level aminoglycoside resistance simultaneously -Carbapenem and colistin-resistant strains in the environment are diverse in clonal backgrounds	[9]
Spain	Sewage water	2013 (*mcr*-1)	90	*mcr*-1 (29 *E. coli*,1 *K. pneumoniae*)	*E. coli* (ST1196/ST632); *K. pneumoniae* (ST526)	Inc2	*bla*_CTX-M-55_ and *bla*_TEM-1_	- *mcr*-1 can act as silent gene thereby evading phenotypic detection and favouring its dissemination -Sewages are huge reservoirs of *mcr*-1 from where it is disseminated to other ecosystems	[81]
Thailand	Wastewaters	2015 (*mcr*-1 and *mcr*-2)	65	*mcr*-1 (2 *E. coli*)	ST5951and ST6624	IncHI2 and IncX4	*bla*_CTX-M-14_	- Diverse carbapenem- and colistin-resistant *E. coli* clones are shared between humans and their immediate environment	[83]
Bangladesh	Urban sludge	-	48	*mcr*-1 (1 *E. coli*)	-	-	-	- First report of *mcr*-1-positive isolate in Bangladeshi environment	[84]
China	Sewage	2015 (*mcr*-1)	9	*mcr*-1 (9 *E. coli* and 2 *Kluyvera ascobarta*), novel *mcr*-1.7 and *mcr*-1.4 (2. *E. coli*)	ST10, ST34, ST48, ST1196 and novel ST7086 and ST7087 (*dnaG*, *int*, *parA*, *nikB*, *traB*, *ydfA*, *ydgA*, *yejO* and *yfjP*)	IncX4, IncI2, IncHI2, IncN and IncP (IS*ApI1*); for chromosome: IS*911*∆,IS*30*∆, *Ec23*∆ and IS*1294*)	*bla*_CTX-M-14_, *bla*_TEM-1b_, *bla*_CTX-M-55_, *bla*_CMY-2_, *bla*_CTX-M-125_, *strA*, *strB*, *aac(3)-IId*, *aadA2*, *aadA1*, *aadA22, aph(30)-Ia*, *aac(3)-Iva)*, *ph(4)-Ia*, *oqxB*, *oqxA, qnRS1*, *fosA*, *lnu(F)*, *mph(A)*, *met(B)*, *cmlA1* and *floR* genes, *sul1*, *sul2*, *sul3*, *tet(M)*, *tet(B)*, *tet(A)*, *dfrA14* and *dfrA12*	– First report of *mcr*-1.4 and *mcr*-1.7 - *mcr*-1 gene variants are carried on chromosome and plasmids in diverse unrelated species	[87]
China	Hospital sewage	2015 (*mcr*-1)	1	*mcr*-1 (*K. pneumoniae*)	ST313 (*tra*, *trb* and *trfA*)	IncP-1 (IS*Apl1* and IS*26*)	*aac(3)-Iva*, *aadA2*, *aph(3′)-Ia, aph(4)-I*, *bla*_TEM-135_ and new *bla*_SHV195_ variant, *floR*, *fosA, oqxA*, *oqxB*, *sul2*, *dfrA12*, *tet(A)* and ORFble	- *mcr*-1-poitive *K. pneumoniae* ST313 can spread through sewage - First report of ORFble gene with undetermined function in environmental isolate	[88]
China	Hospital sewage	2016 (*mcr*-1 and *mcr*-2)	25	*mcr*-1 (9 *E. coli*)	ST7122, ST10, ST410, ST2016, ST349, ST6756 and ST101	IncX4	*bla*_CTX-M-15_, *bla*_CTX-M-3-like_, *bla*_CTX-M-14_ and *bla*_TEM-1_	- First report of *mcr*-1-positive *E. coli* and *bla*_KPC_-carrying *Enterobacter cloacae* in sewage	[89]
China	Hospital sewage	2015 (*mcr*-1)	1	*mcr*-1 (1 *Kluyvera ascobarta*)	*traC*	IncI2 (IS*ApI1*)	*bla*_CTX-M-185_ and *fosA*	**- First report of *mcr*-1-positive *Klyuvera ascorbata* in environmental sample	[90]
Lebanon	Sewer water	*mcr*-1 to *mcr*-8	8	*mcr*-1 (8 *Proteus mirabilis*)	-	-	*bla*_TEM_ and *IntI*1	- First report of *mcr*-1 in *Proteus mirabilis* in sewers highlighting the need for improved sanitation in war refugee camps in Lebanon	[95]

*mcr*: mobile colistin resistance gene; -: no data; Additional resistance traits: resistance factors identified in one *mcr*-positive isolate or pooled factors in more than one *mcr*-positive isolate; Sequence type: all sequence types of *mcr* gene-positive isolates; Plasmid: plasmid types identified in one or pooled *mcr* gene-positive isolates; Inc.: incompatibility; ∆: truncated; IS: insertion sequence.

**Table 3:** Studies reporting plasmid-mediated colistin resistance in isolates from fresh and seawaters.

Country	Source of Isolate	Date of Isolation (*mcr* Gene Assayed)	Number of Isolates Tested for *mcr*	Identified Gene/Variant (Number of Organism)	Sequence Type and/or Phylogroup (Virulence Genes)	Plasmid (Associated Insertion Sequence)	Additional Resistance Traits	Key Points/Conclusion	Reference
China	Well water	2015 (*mcr*-1)	10	*mcr*-1 (2 *E. coli*)	ST48 and ST10	-	*bla*_CTX-M-14_ and *bla*_CTX-M-65_	-*mcr*-1-positive ESBL-producing *E. coli* of ST10 complex and carbapenem-resistant organisms have disseminated into ground water in China highlighting that these waters are sources of resistance to last resort antibiotics	[62]
Brazil	Coastal water at public beaches	2016 (*mcr*-1)	3	*mcr*-1 (3 *E. coli*)	ST1638, ST46 and ST10, and B1 (*iss*, *gad* and *mchF)*	IncI1, ColRNAI, IncX4, IncFIB, IncQ1, IncX4 IncHI2 and IncN.	*bla*_CTX-8_, *bla*_CTX-M-1_, *qnrB19*, *aadA2*, *strA*, *strB*, *sul2*, *bla*_TEM-1B_, *catA1*, *aadA1*, *sul1*, *tetA*, *dfrA1*, *dfrA8* and *tetB*	-Anthropogenic activities results in dissemination of *mcr*-1-carrying *E. coli* to coastal waters in Brazil presenting health danger to users of public beaches	[103]
Italy	Well/Stream	2014–2015 (*mcr*-1)	264	*mcr*-1.2 (1 *E. coli*)	ST10 (*pap2*)	IncX4 and IncX3	*bla*_CTX−M−1_ and *bla*_SHV−1_	-First report on *mcr*-1.2 in ST10 *E. coli* from ground water highlighting the need for improved water treatment protocols and surveillance of resistance genes -Environmental water is a reservoir of antimicrobial resistance genes	[100]
Norway	Water from public beach	2010 (*mcr*-1)	82	*mcr*-1 (2 *E. coli*)	ST10 (A and B1)	IncFII, IncI1, IncFIB, Col156, IncX4 and ColRNAI	*aadA5*, *bla*_CTX-M-1_, *bla*_TEM-1B_, *dfrA17*, *strA*, *strB*, *sul2* and *tet(B)*	-*mcr*- 1 have spread to areas without selection pressure -*mcr*-1-carrying Enterobacteriaceae in Norwegian surface waters emanate from anthropogenic sources	[101]
Malaysia	Water	2014	1	*mcr*-1(1 *E. coli*)	*nikB*	IncI	-	-*mcr*-1 have been in existence more than expected but IS*Apl1*facilitated its spread among bacteria	[105]
Malysia	Pond water	2013	1	*mcr*-1 (*E. coli*)	ST410	IncFII, Incl2, ColRNAI, and IncFIB	*aph(3)-Ia*, *aadA1*, *aadA2*, *strA*, *strB*, *bla*_TEM-1B_, *fosA*, *catA2*, *cmlA1*, *sul3*, *tet(A)*, *tet(34)* and *drfA14*	-*mcr*-1-positive *E. coli* strains are non-clonal and they often carry multiple resistance genes	[106]
Lebanon	Irrigation water	2018 (*mcr*-1 to *mcr*-8)	22	*mcr*-1 (22 *E. coli*)	-	-	*Intl1*, *bla*_TEM_, *bla*_CTX-M_, *bla*_SHV_, *bla*_NDM-1_ and *bla*_OXA-48_	- *mcr*-1 can persist in water matrix -Antimicrobial-resistant organisms in Lebanese irrigation can spread into the Mediterranean Basin	[108]
Lebanon	Sewer/domestic waters	*mcr*-1	36	*mcr*-1 (36 *E. coli*)	-	IncI2 and IncX4	*Intl*1, *bla*_TEM_, *bla*_CTX-M_, *bla*_SHV_, *bla*_OXA-48_ and *bla*_KPC_	-Unsanitary conditions in Lebanon and Syrian war refugee camps facilitate the spread of *mcr*-1	[109]
China	River, lake and fountain water	2017 (*mcr*-1 to *mcr*-7)	5	*mcr*-3.14 (2 *Aeromonas caviae*); *mcr*-3-like4 (1 *A. veronii*, 1 *A. caviae* and 2 *A. bivalvium*); *mcr*-3.18 (1 *A. caviae*)	*hylA* and *dgkA* in *A. caviae*	-	Except in *A. bivalvium*:*bla*_MOX-7_, *aadA1*, *tet(A)*, *sul1*, *bla*_MOX-4_ and *tet(E)*	-First report of *mcr*-3.13 to *mcr*-3.18 -*Aeromonas* which is common in aquatic environment represent important reservoir of *mcr*-3group gene	[110]
China	River/lake/fountain water	*(mcr*-1, and *mcr*-2)	*mcr*-1 (detected in samples)	*mcr*-1 (16 *E. coli*, 1 *Enterobacter cloacae*, *Citrobacter freundii* (2)*, Citrobacter braakii* (2) and *Kebsiella oxytoca* (2)	ST10, ST43, ST101, ST206, ST1638 and ST181	-	*qnrS*, *oqxA*, *oqxB*, *qnrB*, *bla*_CTX-M-15_, *bla*_CTX-M-55_ and *bla*_TEM-1_	-Enrichment broth culture increases the rate of detection of *mcr*-1-carrying organism in water -Responsible use of polymyxins in agriculture and clinical settings curbs the spread of *mcr*-1into the environment	[113]
China	River	2017 (*mcr*-1 to *mcr*-5)	1500	*mcr*-1 (17 *E. coli* and 1 *Enterobacter cloacae*) and *mcr*-3 (2 *Aeromonas veronii* and 4 *A. hydrophila*)	-	-	*bla*_SHV_, *bla*_TEM_, *bla*_CTX−M−9_, *sul1*, *sul2*, *tetM*, *tetA*, *qnrB*, *qnrS*, *oqxAB*, *aac(6′)-Ib-cr*, *rmtA*, *rmtB*, *floR* and *fosA*.	-Sewage is a source of antibiotic resistance genes in urban rivers	[114]
Algeria	Sea water	2016 (*mcr*-1)	246	*mcr*-1.1 (1 *E. coli*), *mcr*-1.5 (1 *E. coli*)	ST23 and ST115	IncI2 and IncHI2A	*aac(3)-IId*, *aadA1*, *aadA2*, *aph(3′)-Ia*, *aph(3)-Ib*, *aph(6)-Id*, *bla*_TEM-1B_, *mph(A)*, *cml*, *sul1*, *sul3*, *tet(A)*, *dfrA*, and *dfrA14*.	-First report of *mcr*-1-carrying *E. coli* ST23 and ST115 in Algerian coast highlighting the global spread of the gene and the need for improved waste water/sewage treatment protocols	[102,115]

*mcr*: mobile colistin resistance gene; -: no data; Additional resistance traits: resistance factors identified in one or pooled *mcr*-positive isolate; Sequence type: all sequence types of *mcr*-positive isolates; Plasmid: plasmid types in one orpooled *mcr*-positive isolate; Inc.: incompatibility; IS: insertion sequence.

**Table 4:** Studies reporting plasmid-mediated colistin resistance in aquaculture environment and aquatic-based foods.

Country	Source of Isolate	Date of Isolation (*mcr* Gene Assayed)	Number of Isolates Tested for *mcr*	Identified Gene/Variant (Number of Organism)	Sequence Type and/or Phylogroup (Virulence Genes)	Plasmid (Associated Insertion Sequence)	Additional Resistance Traits	Key Points/Conclusion	Reference
China	Integrated fish-duck farm	(*mcr*-1 to *mcr*-8)	59	*mcr*-1.1 (7 *E. coli*) and *mcr*-3 (1 *Aeromonas*)	ST48,ST93, ST156, ST162 and ST648 (*usp*, *cdtA*, *set1A* and *hylEclyA*)	IncHI2, IncI2, IncX4, IncP and Incp0111 (IS*ApI1*)	*bla*_TEM-1_	-Aquaculture is a route for transfer of *mcr*-1between humans and animals -First report of *mcr*-8 in aquaculture	[28]
Germany	Fishes	2005–2011 (*mcr*-1 to *mcr*-3)	479	*mcr*-3.6 (1 *Aeromonas allosaccharophila*), *mcr*-3.8 (1 *A. jandei* and 1 *A. hydrophila*) and *mcr*-3.9 (1 *A. hydrophila*)	*dgkA*	IS*Ecp1*, ∆IS*Aeme4*, ∆IS*Aeme5*, IS*50R*, IS*3*, IS*5* and IS*30*)	*bla*_CEPH-A3_, *bla*_FOX-5_, *bla*_OXA-12_, *tet(E)*, *ampH*, *cphA2*, *tet(D)* and *tet(M)*	-First report of *mcr*-3.6 to *mcr*-3.9 highlighting that *mcr*-3 group gene have been present for the past 12 years in Europe	[118]
Norway	Imported sea food	2015 (*mcr*-1)	1	*mcr*-1 (1 *E. coli*)	ST48	IncHI2, IncN and IncX3	*gyrA* mutation, *bla*_TEM-1B_, *dfrA12*, *dfrA15*, *mph(A)*, *sul1*, *sul3*, *tet(A)*, *cmlA1*, *strA*, *strB*, *aadA2*, *aadA1*, *qnrS1* and *qepA*.	-Aquatic-based food trade is a route for transfer of *mcr*-1	[117]
Spain	Mussels	2012–2016	19	*mcr*-1 (1 *Salmonella enterica* Serovar Risen)	ST469 (*invA*)	-	*aac(6′)-Iaa*, *aadA1*, *aadA2*, *bla*_TEM-1B_, *cmlA1*, *sul1*, *sul3*, *tet(A)*, and *dfrA1*	-First report of *mcr*-1-positive *Salmonella* in ready-to-eat mussels highlighting the risk of transmission of *mcr*-1 from Galicia to European markets	[120]
China	Grass carp fish	2016–2017 (*mcr*-1 to *mcr*-5)	190	*mcr*-1 (7 *E. coli*)	ST ST48, ST7508, ST2040 ST7013 and ST156	IncI2, IncP and IncX4	*aadA2*, *aadA1*, *aph(3′)-Ic*, *aac(3)-IId*, *strA*, *strB*, *bla*_TEM-1A_, *bla*_CTX-M-14_, *bla*_CTX-M-55_, *bla*_TEM-176_, *bla*_OXA-10_, *bla*_CARB-2_, *qnrS1*, *oqxB*, *oqxA*, *cmlA1*, *floR*, *arr-2*, *sul2*, *sul3*, *tet(A)*, *tet(M)*, *dfrA14*, *dfrA16*, *fosA3*, *erm(B)*, *mph(A)*, *tet(A)*, and *dfrA17*	-IncP plasmid may facilitate the dissemination of *mcr*-1 gene across various hosts just like Inc2 and IncX4 plasmids	[123]

*mcr*: mobile colistin resistance gene; -: no data; Additional resistance traits: resistance factors identified in one or pooled *mcr*-positive isolate; Sequence type: all sequence types of *mcr* gene-positive isolates; Plasmid: plasmid types identified in one or pooled *mcr*-positive isolates; Inc.: incompatibility; ∆: truncated; IS: insertion sequence.

**Table 5:** Studies reporting plasmid-mediated colistin resistance in isolates from soil/manure ecosystem.

Country	Source of Isolate	Date of Isolation (*mcr* Gene Assayed)	Number of Isolates Tested for *mcr*	Identified Gene/Variant (Number of Organism)	Sequence Type and/or Phylogroup (Virulence Genes)	Plasmid (Associated Insertion Sequence)	Additional Resistance Traits	Key Points/Conclusion	Reference
Estonia	Pig slurry	2011–2014 (*mcr*-1)	141	*mcr*-1 (3 *E. coli*)	*taxA*, *taxB*, *taxC*, *trbM*, *pap2*, *topC*, *hicA*, *hicB*, *parA*, *pilX* operon	IncX4 (IS*26*)	*bla*_CTX-M-1_, *bla*_TEM-1_ and *bla*_AmpC-1_	- Highly mobile plasmids carry *mcr*-1 in genetically diverse strains even from the same environment	[126]
China	Farming soil	2016 (*mcr*-1 to *mcr*-5)	53	*mcr*-1 (6 *E. coli*)	ST2060, ST3014, ST6756 and ST1560 (*pap2*)	InFIB, IncX1, IncHI1, IncFII, IncFIA, pO111 and IncHI2 (IS*Apl1*)	*aph(4)-Ia*, *aacA4*, *aac(3)-IVa*, *aph(3′)-Ia*, *aac(6′)-Ib-cr*, *aadA2*, *bla*_TEM-1B_, *bla*_CTX-M-55_, *fosA*, *mph(A)*, *cmlA1*, *floR*, *catA1*, *catB3*, *AAR-3*, *sul1*, *sul2*, *sul3*, *tet(B)*, tet(M), *dfrA1*, *dfrA12*, *bla*_CTX-M-27_, *QnrS1*, *oqXB*, *oqxA*, *mef(B)*, *aac(3)-IId*, *aacA1*, *aacA2, aph(3′)-IIa*, *aadA5*, *dfrA17*, *aph(4)-Ia*, *bla*_CMY-2_ and *bla*_TEM-18_.	- First report of *mcr*-1-carrying ESBL-producing *E. coli* in soil highlighting the influence of animal manure in transmission of colistin resistance	[127]

*mcr*: mobile colistin resistance gene; -: no data; Additional resistance traits: resistance factors identified in one *mcr*-positive isolate or pooled factors in more than one *mcr*-positive isolate; Sequence type: all sequence types of *mcr*-positive isolates; Plasmid: plasmid types identified in one or pooled *mcr*-positive isolates; Inc.: incompatibility; IS: insertion sequence.

**Table 6:** Studies reporting plasmid-mediated colistin resistance in botanical ecosystems.

Country	Sample (Number) Source of Isolate	Date of Isolation (*mcr* Gene Assayed)	Number of Isolates Tested for *mcr*	Identified Gene/Variant (Number of Organism)	Sequence type and/or Phylogroup (Virulence Genes)	Plasmid (Associated Insertion Sequence)	Additional Resistance Traits	Key Points/Conclusion	Reference
Portugal	Vegetables	2013–2014 (*mcr*-1)	138	*mcr*-1 (1 *E. coli*)	ST1716 (*gad*)	IncHI2, IncI1, IncI2, IncQ, IncP and IncY	Class 2 integrons, *aadA1*, *aph(4)-Ia*, *bla*_TEM-1_, estX-12, *floR*, *sat2*, *strA*, *strB*, *sul2*and *tetA*	-Organic and conventional vegetables are reservoirs of resistance genes, integrons and transposons -First report of class 3 integrons in isolate from vegetable	[133]
China	Vegetables	2015- 2016 (*mcr*-1 and *mcr*-2)	244	*mcr*-1 (7 *E. coli* and 2 *Raulotella ornitholytica*)	ST48, ST2505, ST156, ST795 and ST69	Incx4, IncI2 and IncHI2	*floR*, *bla*_CTX-M-14_, *bla*_CTX-M-55_, *fosA* and *oqxAB*	-Vegetables are source of *mcr*-1-carrying Enterobacteriaceae -Proper animal manure disposal and improved irrigation water warranted	[134]
China	Fruit surfaces	2016 (*mcr*-1 to *mcr*-8)	*mcr* was detected in samples	*mcr*-1 (1 *E. coli* and 1 *K. pneumoniae*)	*E. coli* (ST189) and *K. pneumoniae* (ST422) (*astA*)	*E. coli* (IncHI1 and IncFIA); *K. pneumoniae* (IncHI1 and IncFIB)	*E. coli:aadA2*, *aadA1*, *floR*, *cmlA1*, *sul2*, *sul3*, *tetA*, *tetM*, *dfrA12*, *mdfA*; *K. pneumoniae:bla*_SHV-110_, *qnrS1*, *oqxA*, *oqxB*, *fosA6*, *sul1*, *tetA* and *dfrA1*	-Fruits are direct source of *mcr*-1-carrying Enterobacteriaceae to humans	[138]
China	Vegetables	2017–2018 (*mcr*-1 to *mcr*-8)	*mcr*-1 detected in vegetable samples	*mcr*-1 (*23 E. coli and 1 Enterobacter*)		IncX4, IncI2 and IncHI2	*aph(3′)-Ia*, *aac(3)-IV*, *aph(4)-Ia*, *aadA2*, *aadA1*, *cmlA*, *floR*, *tet(M)*, *fosA3*, *bla*_CTX-M-14_, *sul2*, *sul3* and *mph(A)*	-Dissemination of *mcr*-1 among Enterobactericeae in vegetables is mainly mediated by IncX4 and IncI2 plasmids -*mcr*-1 is often co-carried with *bla*_CTX-M_	[135]
China	Vegetables	2017–2018 (*mcr*-1 to *mcr*-8)	-	*mcr*-1 (2 *E. coli*)	ST156 and ST2847	IncX4 and IncI2	*bla*_NDM-5_ and *bla*_NDM-9_	-First report of concomitant occurrence carriage of *bla*_NDM-5/9_ and *mcr*-1 in isolate from fresh vegetables -Plasmids IncX4 and IncI2 have spread globally	[136]

*mcr*: mobile colistin resistance gene; -: no data; Additional resistance traits: resistance factors identified in one or pooled factors *mcr*-positive isolates; Sequence type: all sequence types of *mcr*-positive isolates; Plasmid: plasmid types identified in one or pooled *mcr*-positive isolates; Inc.: incompatibility; IS: insertion sequence.

**Table 7:** Studies reporting plasmid-mediated colistin resistance in wildlife (mammals, birds and flies).

Country	Source of Isolate	Date of Isolation (*mcr* Gene Assayed)	Number of Organism Isolates Tested for *mcr*	Identified Gene/Variant Detected (Number of Organisms)	Sequence Type and/or Phylogroup (Virulence Genes)	Plasmid (Associated Insertion Sequence)	Additional Resistance Traits	Key Points/Conclusion	Reference
Lithuania	Rectal swabs/faeces of European herring gull (*Larus argentatus*)	2016 (*mcr*-1)	117	*mcr*-1 (1 *E. coli*)	-	IncI	-	-First report of *mcr*-1-positive bacteria in migratory bird highlighting the capacity of these birds to spread the gene to various continents	[140]
Spain	Cloacal swabs of a wild bird (black vulture)	2015- 2016 (*mcr*-1 and *mcr*-2)	94	*mcr*-1 (1 *E. coli*)	ST162	-	*bla*_CIT_	-Eating habits of migratory wild birds facilitates acquisition of multiresistant organisms from human environment and global dissemination of these organisms	[141]
Brazil	Pododermatitic Magellanic penguins (*Spheniscus magenallicus*)	June 2013 (*mcr*-1)	1	*mcr*-1 (1 *E. coli*)	ST10 and A (*gad*)	IncFIB, IncN, IncHI2, IncHI2A, IncI1 and IncX4	*bla*_CTX-M-1_, *aadA1*, *sul2*, *tetA* and *tetB*	- First report of *mcr*-1 in penguins highlighting the role of IncX4 plasmid in global dissemination of *mcr*-1 even to wildlife	[143]
Argentina	Faeces of Kelp gulls (*Larus dominicanus*) (50)	2012	5	*mcr*-1 (5 *E. coli*)	ST744 (4 strains), ST101(1 strain)	IncI2 (IS*ApI1*)	*bla_C_*_TX-M-14_ and *bla*_CTX-M-2_	-First report of *mcr*-1 in Kelp gulls highlighting the capacity of these migratory water birds to disseminate the gene globally	[144]
Algeria	Stool of a Barbary macaques monkey (*Macaca sylvanus*) (86)	2016 (*mcr*-1	1	*mcr*-1 (1 *E. coli*)	ST405 (*fyuA*)	-	*bla*_CTX-M-15_, *bla*_TEM-1_ and *qnrB19*	-First report of *mcr*-1 in monkeys -Wild animals can disseminate multidrug-resistant organisms in places frequented by people and in other ecosystems	[55]
China	Flies/faecal swabs/nest swabs of wild birds	2014–2015 (*mcr*-1)	245	*mcr*-1 (37 Enterobacteriaceae); *mcr*-1 detected in samples	-	-	-	-Carbapenem- and colistin-resistant Enterobacteriaceae have disseminated into diverse environment in China	[86]
Pakistan	Cloacal swab of an Eurasian coot (*Fulica tra*)	2014 (*mcr*-1)	1	*mcr*-1 (*E. coli*)	ST345	IncI2	*bla*_CTX-M-15_	-First report of *mcr*-1 in wildlife in Asia highlighting the presence of this gene in environment without selective pressure -Migratory wild bird can disseminate *mcr*-1 from Asia to other continents	[145]
China	Flies in animal farms	2016 (*mcr*-1)	52	*mcr*-1 (*E. cloacae*) and *mcr*-1.3 (1 *Raulotella planticola*)	-	IncI2 and IncX4 (IS*ApI1*)	ESBL genes	-First report of *mcr*-1-positive *Raulotella planticola* -Uncommon species can transmit *mcr*-1 to common enterobacterial species in which COL resistance is enhanced	[149]
Thailand	Blow flies (*Chrysomya megacephala*)	2019 (*mcr*-1)	-	*mcr*-1 (34 *E. coli* and 17 *K. pneumoniae*); *mcr*-8 (17 *K. pneumoniae*)	*E. coli* (ST10, ST648, ST549, ST58, ST181, ST218, ST201, ST162, ST457, ST1244, ST2345, ST2705 and ST5487); *K. pneumoniae*: (ST34); *hicAB*, ehAB, eivA,C,E,F,G,I,J, entA,B,C,E,F,S, epaO,P,Q,S,H,I,JkfuABC, *iro*E, ureABCDEFG, ycfM g, and many others	IncX4, IncHI1B, IncHI1A and IncHI1A-HI1B	*E. coli*: *aac(3′)-IId*, *aph (3′)-Ib*, *aph(6)-Id*, *aph(3′)-Ia*, *aadA2*, *aadA1*, *aadA17*, *bla*_TEM-1b_, *bla*_CTXM-14_, *bla*_CTXM-15_, *mdfA*, *InuF*, *mefB*, *tetA*, *tetB*, *tetM*, *cmlA*, *catA2*, *floR*, *qnrS1*, *dfrA12*, *sul2* and *sul3*; *K. pneumoniae*: *qnrS1*, *bla*_TEM-1b_, *tetA*, *bla*_SHV-40_ and *fosA*	- Flies can disseminate highly virulent multidrug-resistant organisms especially in low- and middle-income countries with unsanitary conditions	[152]

*mcr*: mobile colistin resistance gene; -: no data; Additional resistance traits: resistance factors identified in one or pooled *mcr*-positive isolate; Sequence type: comprise all sequence types of *mcr* gene-positive isolates; Plasmid: plasmid types identified in one or pooled *mcr*-positive isolate; Inc.: incompatibility; IS: insertion sequence.

**Table 8:** Studies reporting plasmid-mediated colistin resistance in isolates from multiple environmental ecosystems.

Country	Source of Isolate	Date of Isolation (*mcr* Gene Assayed)	Number of Isolates Tested for *mcr*	Identified Gene/Variant (Number of Organism)	Sequence Type and/or Phylogroup (Virulence Genes)	Plasmid (Associated Insertion Sequence)	Additional Resistance Traits	Key Points/Conclusion	Reference
Germany	Farm boot, manure, barn flies and barn dog faeces	2011–2012 (*mcr*-1)	25, 3, 1 and 5 from boot swabs, flies, barn dog and manure, respectively	*mcr*-1 (2, 3, 1 and 1 from boot swabs, manure, stable fly, and barn dog, respectively	ST10, ST1140, ST5281, ST1011 and ST342	IncX4, IncI1,IncFII, IncQ1,	*strA*-like, *strB*-like, *bla*_TEM-1B-like_*bla*_CTX-M-1_, *tet(A)*, *aadA1*, *aadA2*, *aad5*, *aph3-like*I, *aph(30)-Ia*-like, *bla*_TEM1B_, *cmlA1*-like, *dfrA1*, *dfrA14*-like, *sul2*, *sul3* and *ul2-like*	-First report of *mcr*-1 in environment close to pig farms -*mcr*-1can be transmitted by diverse routes, including flies feeding on animal manure	[65]
China	Fences and water from swine farm	1999- 2015 (*mcr*-1)	*mcr*-1 detected in samples	*mcr*-1(2 *E. coli* from fence and 1 *E. coli* from water)	-	-	-	-Development of real-time PCR conjugated probes for detection of *mcr*-1 in samples -*mcr*-1 is prevalent among human and animal population in China highlighting first report on detection of *mcr*-1 in pets	[68]
Switzerland	Lake/river water and vegetables	2012 -2014 (*mcr*-1)	74 (water), 60 (vegetable)	*mcr*-1 (1 *E. coli* from water, 2 *E. coli* from vegetable)	ST359/B1, ST167/A, and ST4683/B1	-	*bla*_CTX-M-55_ and *bla*_CTX-M-65_	-Plant-based food trade is a route for dissemination of colistin-resistant Enterobacteriaceae	[99]
Bangladesh	Flies (*Musca domestica*) and pond water	2018 (*mcr*-3)	52	*mcr*-3 (1 *E. coli* from water, and 4 *E. coli* from flies)	-	-	-	-First report of *mcr*-3-positive carbapenem-resistant *E. coli* in Bangladesh	[107]
China	River water and egret faeces	2015 (*mcr*-1)	6	*mcr*-1 (1 *E. coli* from water and 2 *E. coli* from egret faeces)	-	-	*tetL*, *tetO*, *aac6ib*, *aadA*, *bla*_CMY-2_, *bla*_CTX-M-14_, *IntI* and *tnpA-1*	-First report of simultaneous detection of *mcr*-1in egrets and their habitat highlighting the role of migratory water birds in contamination of the environment	[111]
Algeria	Soil/manure and irrigation water	2016–2018 *(mcr*-1 to *mcr*-5)	103	*mcr*-1 (6 *E. coli*) and *mcr*-3 (2 *E. coli*)	ST405, ST10, ST345 and ST155	-	*bla*_TEM-12_	First report of *mcr*-3 in environmental isolates in Algiers Animal manure is a source for transfer of *mcr* to soil and irrigation water	[131]

*mcr*: mobile colistin resistance gene; -: no data; Additional resistance traits: resistance factors identified in one or pooled *mcr*-positive isolates; Sequence type: all sequence types of *mcr*-positive isolates; Plasmid: plasmid types identified in one or pooled *mcr*-positive isolates; Inc.: incompatibility; IS: insertion sequence.

## References

[1] Ma K., Feng Y., Zongid Z. (2018). Fitness cost of a mcr-1-carrying IncHI2 plasmid. PLoS ONE.

[2] Wang R., van Dorp L., Shaw L.P., Bradley P., Wang Q., Wang X., Jin L., Zhang Q., Liu Y., Rieux A. (2018). The global distribution and spread of the mobilized colistin resistance gene mcr-1. Nat. Commun..

[3] Carretto E., Brovarone F., Russello G., Nardini P., El-Bouseary M.M., Aboklaish A.F., Walsh T.R., Tyrrell J.M. (2018). Clinical Validation of SensiTest Colistin, a Broth Microdilution-Based Method to Evaluate Colistin MICs. J. Clin. Microbiol..

[4] Liu Y.-Y., Wang Y., Walsh T.R., Yi L.-X., Zhang R., Spencer J., Doi Y., Tian G., Dong B., Huang X. (2016). Emergence of plasmid-mediated colistin resistance mechanism MCR-1 in animals and human beings in China: A microbiological and molecular biological study. Lancet Infect. Dis..

[5] Mcgann P., Snesrud E., Maybank R., Corey B., Ong A.C., Clifford R., Hinkle M., Whitman T., Lesho E., Schaecher K.E. (2016). *Escherichia coli* Harboring mcr-1 and bla CTX-M on a Novel IncF Plasmid: First Report of mcr-1 in the United States. Antimicrob. Agents Chemother..

[6] Abuoun M., Stubberfield E.J., Duggett N.A., Kirchner M., Dormer L., Nunez-Garcia J., Randall L.P., Lemma F., Crook D.W., Teale C. (2017). mcr-1 and mcr-2 (mcr-6.1) variant genes identified in Moraxella species isolated from pigs in Great Britain from 2014 to 2015. J. Antimicrob. Chemother..

[7] Partridge S.R., Di Pilato V., Doi Y., Feldgarden M., Haft D.H., Klimke W., Kumar-Singh S., Liu J.-H., Malhotra-Kumar S., Prasad A. (2018). Proposal for assignment of allele numbers for mobile colistin resistance (mcr) genes. J. Antimicrob. Chemother..

[8] Carroll L.M., Gaballa A., Guldimann C., Sullivan G., Henderson L.O., Wiedmann M. (2019). Identification of Novel Mobilized Colistin Resistance Gene mcr-9 in a Multidrug-Resistant, Colistin-Susceptible Salmonella enterica Serotype Typhimurium Isolate. MBio.

[9] Long H., Feng Y., Ma K., Liu L., Mcnally A., Zong Z. (2019). The co-transfer of plasmid-borne colistin-resistant genes mcr-1 and mcr-3.5, the carbapenemase gene bla NDM-5 and the 16S methylase gene rmtB from *Escherichia coli* OPEN. Sci. Rep..

[10] Son S.J., Huang R., Squire C.J., Leung I.K.H. (2019). MCR-1: A promising target for structure-based design of inhibitors to tackle polymyxin resistance. Drug Discov. Today.

[11] Gaze W.H., Krone S.M., Larsson D.G.J., Li X.-Z., Robinson J.A., Simonet P., Smalla K., Timinouni M., Topp E., Wellington E.M. (2013). Influence of Humans on Evolution and Mobilization of Environmental Antibiotic Resistome. Emerg. Infect. Dis..

[12] Shen Y., Yin W., Liu D., Shen J., Wang Y., Shen C.Y., Karen Bush E. (2018). Reply to Cabello et al., “Aquaculture and mcr Colistin Resistance Determinants”. MBio.

[13] Lim L.M., Ly N., Anderson D., Yang J.C., Macander L., Iii A.J., Forrest A., Bulitta J.B., Tsuji B.T. (2010). Resurgence of Colistin: A Review of Resistance, Toxicity, Pharmacodynamics, and Dosing HHS Public Access. Pharmacother. J. Hum. Pharmacol. Drug Ther..

[14] Skov R.L., Monnet D.L. (2016). Plasmid-mediated colistin resistance (mcr-1 gene): Three months later, the story unfolds. Euro Surveill..

[15] Liu Y., Liu J.-H. (2018). Monitoring Colistin Resistance in Food Animals, An Urgent Threat. Expert Rev. Anti. Infect. Ther..

[16] Al-tawfiq J.A., Laxminarayan R., Mendelson M. (2017). How should we respond to the emergence of plasmid-mediated colistin resistance in humans and animals?. Int. J. Infect. Dis..

[17] Sun J., Zeng X., Li X.-P., Liao X.-P., Liu Y.-H., Lin J. (2018). Plasmid-mediated colistin resistance in animals: Current status and future directions. Anim. Heal. Res. Rev..

[18] Nang S.C., Li J., Velkov T. (2019). The rise and spread of *mcr* plasmid-mediated polymyxin resistance. Crit. Rev. Microbiol..

[19] Sekyere J.O. (2019). Mcr colistin resistance gene: A systematic review of current diagnostics and detection methods. Microbiologyopen.

[20] He T., Wang R., Liu D., Walsh T.R., Zhang R., Lv Y., Ke Y., Ji Q., Wei R., Liu Z. (2019). Emergence of plasmid-mediated high-level tigecycline resistance genes in animals and humans. Nat. Microbiol..

[21] O’Neill J. (2014). Antimicrobial Resistance: Tackling a Crisis for the Health and Wealth of Nations.

[22] Fletcher S. (2015). Understanding the contribution of environmental factors in the spread of antimicrobial resistance. Environ. Health Prev. Med..

[23] Martinez J.L., Luis Balcazar J., Singer A.C., Shaw H., Rhodes V., Hart A. (2016). Review of Antimicrobial Resistance in the Environment and Its Relevance to Environmental Regulators. Front. Microbiol..

[24] Bengtsson-Palme J., Kristiansson E., Larsson D.G.J. (2018). Environmental factors influencing the development and spread of antibiotic resistance. FEMS Microbiol. Rev..

[25] Tiedje J.M., Fang W., Manaia C.M., Virta M., Sheng H., Liping M.A., Tong Z., Topp E. (2019). Antibiotic Resistance Genes in the Human-Impacted Environment: A One Health Perspective. Pedosphere.

[26] United Nations Environment Programme (UNEP) Antimicrobial Resistance from Environmental Pollution Among Biggest Emerging Health Threats, Says UN Environment|UN Environment. https://www.unenvironment.org/news-and-stories/press-release/antimicrobial-resistance-environmental-pollution-among-biggest.

[27] Zhang H., Wei W., Huang M., Umar Z., Feng Y., Zhang H., Wei W., Huang M., Umar Z., Feng Y. (2019). Definition of a Family of Nonmobile Colistin Resistance (NMCR-1) Determinants Suggests Aquatic Reservoirs for MCR-4. Adv. Sci..

[28] Shen Y., Lv Z., Yang L., Liu D., Ou Y., Xu C., Liu W., Yuan D., Hao Y., He J. (2019). Integrated aquaculture contributes to the transfer of mcr-1 between animals and humans via the aquaculture supply chain. Environ. Int..

[29] Caselli E., D’Accolti M., Soffritti I., Piffanelli M., Mazzacane S. (2018). Spread of *mcr-1*–Driven Colistin Resistance on Hospital Surfaces, Italy. Emerg. Infect. Dis..

[30] Rodriguez-Mozaz S., Chamorro S., Marti E., Huerta B., Gros M., Sànchez-Melsió A., Borrego C.M., Barceló D., Balcázar J.L. (2015). Occurrence of antibiotics and antibiotic resistance genes in hospital and urban wastewaters and their impact on the receiving river. Water Res..

[31] Dalmolin T.V., De Lima-Morales D., Barth A.L. (2018). Plasmid-mediated Colistin Resistance: What Do We Know?. J. Infect..

[32] Harbottle H., Thakur S., Zhao S., White D.G. (2007). Genetics of Antimicrobial Resistance. Anim. Biotechnol..

[33] Van Hoek A.H.A.M., Mevius D., Guerra B., Mullany P., Roberts A.P., Aarts H.J.M. (2011). Acquired antibiotic resistance genes: An overview. Front. Microbiol..

[34] Partridge S.R., Kwong S.M., Firth N., Jensen S.O. (2018). Mobile Genetic Elements Associated with Antimicrobial Resistance. Clin. Microbiol. Rev..

[35] Mcmanus M.C. (1997). Mechanisms of bacterial resistance to antimicrobial agents. Am. J. Health Syst. Pharm..

[36] DiRita V.J., Cary Engleberg N., Heath A., Miller A., Adam Crawford J., Yu R. (2000). Virulence gene regulation inside and outside. Philos. Trans. R. Soc. Lond. Ser. B Biol. Sci..

[37] Carattoli A. (2009). Resistance Plasmid Families in Enterobacteriaceae. Antimicrob. Agents Chemother..

[38] Jain A., Srivastava P. (2013). Broad host range plasmids. FEMS Microbiol. Lett..

[39] Sultan I., Rahman S., Jan A.T., Siddiqui M.T., Mondal A.H., Haq Q.M.R. (2018). Antibiotics, Resistome and Resistance Mechanisms: A Bacterial Perspective. Front. Microbiol..

[40] Bennett P.M. (2008). Plasmid encoded antibiotic resistance: Acquisition and transfer of antibiotic resistance genes in bacteria. Br. J. Pharmacol..

[41] Dolejska M., Papagiannitsis C.C. (2018). Plasmid-mediated resistance is going wild. Plasmid.

[42] Rozwandowicz M., Brouwer M., Fischer J., Wagenaar J.A., Gonzalez-Zorn B., Guerra B., Mevius D.J., Hordijk J. (2018). Plasmids carrying antimicrobial resistance genes in Enterobacteriaceae. J. Antimicrob. Chemother..

[43] Maillard J.-Y. (2018). Resistance of Bacteria to Biocides. Microbiol. Spectr..

[44] Thanner S., Drissner D., Walsh F. (2016). Antimicrobial Resistance in Agriculture. MBio.

[45] Heaton J.C., Jones K. (2008). Microbial contamination of fruit and vegetables and the behaviour of enteropathogens in the phyllosphere: A review. J. Appl. Microbiol..

[46] Hö C.S., Tetens J.L., Schwaiger K. (2018). Unraveling the Role of Vegetables in Spreading Antimicrobial-Resistant Bacteria: A Need for Quantitative Risk Assessment. Foodborne Pathog. Dis..

[47] Cabello F.C., Godfrey H.P. (2018). Aquaculture, Exaptation, and the Origin of mcr-Positive Colistin Resistance. J. Antimicrob. Chemother..

[48] Cabello F.C., Tomova A., Ivanova L., Godfrey H.P., Bratislava I., Bratislava S. (2017). Aquaculture and mcr Colistin Resistance Determinants. MBio.

[49] Feudi C., Curcio L., Orsini S., Luppi A., Pezzotti G., Magistrali C. (2017). Novel plasmid-mediated colistin resistance mcr-4 gene in Salmonella and *Escherichia coli*, Italy 2013, Spain and Belgium, 2015 to 2016. Eurosurveillance.

[50] Radhouani H., Silva N., Poeta P., Torres C., Correia S., Igrejas G. (2014). Potential impact of antimicrobial resistance in wildlife, environment and human health. Front. Microbiol..

[51] Carroll D., Wang J., Fanning S., McMahon B.J. (2015). Antimicrobial Resistance in Wildlife: Implications for Public Health. Zoonoses Public Health.

[52] Arnold K.E., Williams N.J., Bennett M. (2016). Pathogen biology “Disperse abroad in the land”: The role of wildlife in the dissemination of antimicrobial resistance. Biol. Lett..

[53] Vittecoq M., Godreuil S., Prugnolle F., Durand P., Brazier L., Renaud N., Arnal A., Aberkane S., Jean-Pierre H., Gauthier-Clerc M. (2016). Antimicrobial resistance in wildlife. J. Appl. Ecol..

[54] Wang J., Ma Z.-B., Zeng Z.-L., Yang X.-W., Huang Y., Liu J.-H. (2017). The role of wildlife (wild birds) in the global transmission of antimicrobial resistance genes. Zool. Res..

[55] Bachiri T., Lalaoui R., Bakour S., Allouache M., Belkebla N., Rolain J.M., Touati A. (2018). First Report of the Plasmid-Mediated Colistin Resistance Gene *mcr-1* in *Escherichia coli* ST405 Isolated from Wildlife in Bejaia, Algeria. Microb. Drug Resist..

[56] Anyanwu M.U., Chah K.F. (2016). Antibacterial Resistance in African Catfish Aquaculture: A Review. Not. Sci. Biol..

[57] Onwugamba F.C., Fitzgerald J.R., Rochon K., Guardabassi L., Alabi A., Kühne S., Grobusch M.P., Schaumburg F. (2018). The role of ‘filth flies’ in the spread of antimicrobial resistance. Travel Med. Infect. Dis..

[58] Wetzker W., Pfeifer Y., Wolke S., Haselbeck A., Leistner R., Kola A., Gastmeier P., Salm F., Wetzker W., Pfeifer Y. (2019). Extended-Spectrum Beta-Lactamase (ESBL)-Producing *Escherichia coli* Isolated from Flies in the Urban Center of Berlin, Germany. Int. J. Environ. Res. Public Health.

[59] Bonnedahl J., Järhult J.D. (2014). Antibiotic resistance in wild birds. Upsala J. Med. Sci..

[60] Collignon P., McEwen S., Collignon P.J., McEwen S.A. (2019). One Health—Its Importance in Helping to Better Control Antimicrobial Resistance. Trop. Med. Infect. Dis..

[61] Rousham E.K., Unicomb L., Islam M.A. (2018). Human, animal and environmental contributors to antibiotic resistance in low-resource settings: Integrating behavioural, epidemiological and One Health approaches. Proc. R. Soc. B.

[62] Sun P., Bi Z., Nilsson M., Zheng B., Berglund B., Lundborg C.S., Börjesson S., Li X., Chen B., Yin H. (2017). Occurrence of bla KPC-2, bla CTX-M, and mcr-1 in Enterobacteriaceae from Well Water in Rural China. Antimicrob. Agents Chemother..

[63] Mcewen S.A., Fedorka-Cray P.J. (2002). Antimicrobial Use and Resistance in Animals. Clin. Infect. Dis..

[64] Mohsin M., Raza S. (2017). Comment on “The role of wildlife (wild birds) in the global transmission of antimicrobial resistance genes”. Zool. Res..

[65] Guenther S., Falgenhauer L., Semmler T., Chakraborty T., Roesler U., Roschanski N. (2017). Environmental emission of multiresistant *Escherichia coli* carrying the colistin resistance gene mcr-1 from German swine farms. J. Antimicrob. Chemother..

[66] Lekunberri I., Balcázar J.L., Borrego C.M. (2017). Detection and quantification of the plasmid-mediated mcr-1 gene conferring colistin resistance in wastewater. Int. J. Antimicrob. Agents.

[67] Shen C., Feng S., Chen H., Dai M., Paterson D.L., Zheng X., Wu X., Zhong L.-L., Liu Y., Xia Y. (2018). Transmission of *mcr-1*-Producing Multidrug-resistant Enterobacteriaceae in Public Transportation in Guangzhou, China. Clin. Infect. Dis..

[68] Chen X., Zhao X., Che J., Xiong Y., Xu Y., Zhang L., Lan R., Xia L., Walsh T.R., Xu J. (2017). Detection and dissemination of the colistin resistance gene, mcr-1, from isolates and faecal samples in China. J. Med. Microbiol..

[69] Langford B.J., Schwartz K.L. (2018). Bringing home unwelcome souvenirs: Travel and drug-resistant bacteria. Can. Commun. Dis. Rep..

[70] Riley L.W. (2014). Pandemic lineages of extraintestinal pathogenic *Escherichia coli*. Clin. Microbiol. Infect..

[71] Maluta R.P., Logue C.M., Casas M., Meng T., Guastalli E. (2014). Overlapped Sequence Types (STs) and Serogroups of Avian Pathogenic (APEC) and Human Extra-Intestinal Pathogenic (ExPEC) *Escherichia coli* Isolated in Brazil. PLoS ONE.

[72] Dutta A., Barua H., Jalal M., Dhar P., Biswas S., Biswas P. (2018). An investigation of plasmid-mediated colistin resistance mechanism, MCR in *Escherichia coli* of human, veterinary and environmental origin in Bangladesh. Int. J. Infect. Dis..

[73] Manges A.R., Geum H.M., Guo A., Edens T.J., Fibke C.D., Pitout J.D.D. (2019). Global Extraintestinal Pathogenic *Escherichia coli* (ExPEC) Lineages. Clin. Microbiol. Rev..

[74] Roschanski N., Falgenhauer L., Grobbel M., Guenther S., Kreienbrock L., Imirzalioglu C., Roesler U. (2017). Retrospective survey of mcr-1 and mcr-2 in German pig-fattening farms, 2011–2012. Int. J. Antimicrob. Agents.

[75] Olaitan A.O., Morand S., Rolain J.-M. (2014). Mechanisms of polymyxin resistance: Acquired and intrinsic resistance in bacteria. Front. Microbiol..

[76] Aghapour Z., Gholizadeh P., Ganbarov K., Bialvaei A.Z., Mahmood S.S., Tanomand A., Yousefi M., Asgharzadeh M., Yousefi B., Samadi Kafil H. (2019). Molecular mechanisms related to colistin resistance in Enterobacteriaceae. Infect. Drug Resist..

[77] Van Duijkeren E., Schink A.-K., Roberts M.C., Wang Y., Schwarz S. (2018). Mechanisms of Bacterial Resistance to Antimicrobial Agents. Microbiol. Spectr..

[78] Lood R., Ertürk G., Mattiasson B. (2017). Revisiting antibiotic resistance spreading in wastewater treatment plants—Bacteriophages as a much neglected potential transmission vehicle. Front. Microbiol..

[79] Hembach N., Schmid F., Alexander J., Hiller C., Rogall E.T., Schwartz T. (2017). Occurrence of the mcr-1 Colistin Resistance Gene and other Clinically Relevant Antibiotic Resistance Genes in Microbial Populations at Different Municipal Wastewater Treatment Plants in Germany. Front. Microbiol..

[80] Kneis D., Berendonk T.U., Heß S. (2019). High prevalence of colistin resistance genes in German municipal wastewater. Sci. Total Environ..

[81] Ovejero C.M., Delgado-Blas J.F., Calero-Caceres W., Muniesa M., Gonzalez-Zorn B. (2017). Spread of mcr-1-carrying Enterobacteriaceae in sewage water from Spain. J. Antimicrob. Chemother..

[82] Lerminiaux N.A., Cameron A.D.S. (2018). Horizontal transfer of antibiotic resistance genes in clinical environments. Can. J. Microbiol..

[83] Runcharoen C., Raven K.E., Reuter S., Kallonen T., Paksanont S., Thammachote J., Anun S., Blane B., Parkhill J., Peacock S.J. (2017). Whole genome sequencing of ESBL-producing *Escherichia coli* isolated from patients, farm waste and canals in Thailand. Genome Med..

[84] Islam A., Rahman Z., Monira S., Rahman M.A., Camilli A., George C.M., Ahmed N., Alam M. (2017). Colistin resistant *Escherichia coli* carrying mcr-1 in urban sludge samples: Dhaka, Bangladesh. Gut Pathog..

[85] Chen K., Wai-Chi Chan E., Xie M., Ye L., Dong N., Chen S., Kaichao C., Edward Wai-Chi C., Miaomiao X., Liangwei Y. (2017). Widespread distribution of mcr-1-bearing bacteria in the ecosystem, 2015 to 2016. Eurosurveillance.

[86] Wang Y., Zhang R., Li J., Wu Z., Yin W., Schwarz S., Tyrrell J.M., Zheng Y., Wang S., Shen Z. (2017). Comprehensive resistome analysis reveals the prevalence of NDM and MCR-1 in Chinese poultry production. Nat. Microbiol..

[87] Zhao F., Feng Y., Lü X., McNally A., Zong Z. (2017). Remarkable Diversity of *Escherichia coli* Carrying mcr-1 from Hospital Sewage with the Identification of Two New mcr-1 Variants. Front. Microbiol.

[88] Zhao F., Feng Y., Lü X., Mcnally A., Zong Z. (2017). IncP Plasmid Carrying Colistin Resistance Gene mcr-1 in Klebsiella pneumoniae from Hospital Sewage. Antimicrob. Agents Chemother..

[89] Jin L., Wang R., Wang X., Wang Q., Zhang Y., Yin Y., Wang H. (2018). Emergence of mcr-1 and carbapenemase genes in hospital sewage water in Beijing, China. J. Antimicrob. Chemother..

[90] Zhao F., Zong Z. (2016). Kluyvera ascorbata Strain from Hospital Sewage Carrying the mcr-1 Colistin Resistance Gene. Antimicrob. Agents Chemother..

[91] Roer L., Overballe-Petersen S., Hansen F., Schønning K., Wang M., Røder B.L., Hansen D.S., Justesen U.S., Andersen L.P., Fulgsang-Damgaard D. (2018). *Escherichia coli* Sequence Type 410 Is Causing New International High-Risk Clones. Msphere.

[92] Liu L., Feng Y., Zhang X., Mcnally A., Zong Z. (2017). New Variant of mcr-3 in an Extensively Drug-Resistant *Escherichia coli* Clinical Isolate Carrying mcr-1 and bla NDM-5. J. Antimicrob. Chemother..

[93] Yang R.-S., Feng Y., Lv X.-Y., Duan J.-H., Chen J., Fang L.-X., Xia J., Liao X.-P., Sun J., Liu Y.-H. (2016). Emergence of NDM-5- and MCR-1-Producing *Escherichia coli* Clones ST648 and ST156 from a Single Muscovy Duck (*Cairina moschata*). Antimicrob. Agents Chemother..

[94] He T., Wei R., Zhang L., Sun L., Pang M., Wang R., Wang Y. (2017). Characterization of NDM-5-positive extensively resistant *Escherichia coli* isolates from dairy cows. Vet. Microbiol..

[95] Alhaj Sulaiman A.A., Kassem I.I. (2019). First report of the plasmid-borne colistin resistance gene (mcr-1) in Proteus mirabilis isolated from domestic and sewer waters in Syrian refugee camps. Travel Med. Infect. Dis..

[96] Fernandes M., Moura Q., Sartori L., Silva K., Cunha M., Esposito F., Lopes R., Otutumi L., Gonçalves D., Dropa M. (2016). Silent dissemination of colistin-resistant *Escherichia coli* in South America could contribute to the global spread of the mcr-1 gene. Eurosurveillance.

[97] Delgado-Blas J.F., Ovejero C.M., Abadia-Patiño L., Gonzalez-Zorn B. (2016). Coexistence of mcr-1 and bla NDM-1 in *Escherichia coli* from Venezuela. J. Antimicrob. Chemother..

[98] Baquero F., Martínez J.L., Cantón R. (2008). Antibiotics and antibiotic resistance in water environments. Curr. Opin. Biotechnol..

[99] Zurfuh K., Poirel L., Nordmann P., Nüesch-Inderbinen M., Hächler H., Stephan R. (2016). Occurrence of the Plasmid-Borne mcr-1 Colistin Resistance Gene in Extended-Spectrum-Lactamase-Producing Enterobacteriaceae in River Water and Imported Vegetable Samples in Switzerland. Antimicrob. Agents Chemother..

[100] Caltagirone M., Nucleo E., Spalla M., Zara F., Novazzi F., Marchetti V.M., Piazza A., Bitar I., De Cicco M., Paolucci S. (2017). Occurrence of Extended Spectrum β-Lactamases, KPC-Type, and MCR-1.2-Producing Enterobacteriaceae from Wells, River Water, and Wastewater Treatment Plants in Oltrepò Pavese Area, Northern Italy. Front. Microbiol..

[101] Jørgensen S.B., Søraas A., Arnesen L.S., Leegaard T., Sundsfjord A., Jenum P.A. (2017). First environmental sample containing plasmid-mediated colistin-resistant ESBL-producing *Escherichia coli* detected in Norway. APMIS.

[102] Drali R., Berrazeg M., Zidouni L.L., Hamitouche F., Abbas A.A., Deriet A., Mouffok F. (2018). Emergence of mcr-1 plasmid-mediated colistin-resistant *Escherichia coli* isolates from seawater. Sci. Total Environ..

[103] Fernandes M.R., Sellera F.P., Esposito F., Sabino C.P., Cerdeira L., Lincopan N. (2017). Colistin-Resistant mcr-1-Positive *Escherichia coli* on Public Beaches, an Infectious Threat Emerging in Recreational Waters. Antimicrob. Agents Chemother..

[104] Bartley P.S., Domitrovic T.N., Moretto V.T., Santos C.S., Ponce-Terashima R., Reis M.G., Barbosa L.M., Blanton R.E., Bonomo R.A., Perez F. (2019). Antibiotic Resistance in Enterobacteriaceae from Surface Waters in Urban Brazil Highlights the Risks of Poor Sanitation. Am. J. Trop. Med. Hyg..

[105] Petrillo M., Angers-Loustau A., Kreysa J. (2016). Possible genetic events producing colistin resistance gene mcr. Lancet Infect. Dis..

[106] Yu C.Y., Ang G.Y., Chin P.S., Ngeow Y.F., Yin W.-F., Chan K.-G. (2016). Emergence of mcr-1-mediated colistin resistance in *Escherichia coli* in Malaysia. Int. J. Antimicrob. Agents.

[107] Sobur A.M., Ievy S., Haque Z.F., Nahar A., Zaman S.B., Rahman M.T. (2019). Emergence of colistin-resistant *Escherichia coli* in poultry, house flies, and pond water in Mymensingh, Bangladesh. J. Adv. Vet. Anim. Res..

[108] Hmede Z., Sulaiman A.A.A., Jaafar H., Kassem I.I. (2019). Emergence of plasmid-borne colistin resistance gene mcr-1 in multidrug-resistant *Escherichia coli* isolated from irrigation water in Lebanon. Int. J. Antimicrob. Agents.

[109] Sulaiman A.A.A., Kassem I.I. (2019). First report on the detection of the plasmid-borne colistin resistance gene mcr-1 in multi-drug resistant *E. coli* isolated from domestic and sewer waters in Syrian refugee camps in Lebanon. Travel Med. Infect. Dis..

[110] Shen Y., Xu C., Sun Q., Schwarz S., Ou Y., Yang L., Huang Z., Eichhorn I., Walsh T.R., Wang Y. (2018). Prevalence and Genetic Analysis of mcr-3-Positive Aeromonas Species from Humans, Retail Meat, and Environmental Water Samples. Antimicrob. Agents Chemother..

[111] Wu J., Huang Y., Rao D., Zhang Y., Yang K. (2018). Evidence for Environmental Dissemination of Antibiotic Resistance Mediated by Wild Birds. Front. Microbiol..

[112] Chen Y., Chen X., Zheng S., Yu F., Kong H., Yang Q., Cui D., Chen N., Lou B., Li X. (2014). Serotypes, genotypes and antimicrobial resistance patterns of human diarrhoeagenic *Escherichia coli* isolates circulating in southeastern China. Clin. Microbiol. Infect..

[113] Zhou H.-W., Zhang T., Ma J.-H., Fang Y., Wang H.-Y., Huang Z.-X., Wang Y., Wu C., Chen G.-X. (2017). Occurrence of Plasmid- and Chromosome-Carried mcr-1 in Waterborne Enterobacteriaceae in China. Antimicrob. Agents Chemother..

[114] Tuo H., Yang Y., Tao X., Liu D., Li Y., Xie X., Li P., Gu J., Kong L., Xiang R. (2018). The Prevalence of Colistin Resistant Strains and Antibiotic Resistance Gene Profiles in Funan River, China. Front. Microbiol..

[115] Berrazeg M., Deriet A., De Keersmaecker S.C.J., Verhaegen B., Vanneste K., Botteldoorn N., Roosens N.H.C., Mouffok F., Drali R. (2019). Whole-Genome Sequencing of Multidrug-Resistant *Escherichia coli* Strains Harboring the mcr-1 Gene, Isolated from Seawater of the Algiers Coast in Algeria. Microbiol. Resour. Announc..

[116] Dandachi I., Fayad E., Sleiman A., Daoud Z., Rolain J.-M. (2019). Dissemination of Multidrug-Resistant and mcr-1 Gram-Negative Bacilli in Broilers, Farm Workers, and the Surrounding Environment in Lebanon. Microb. Drug Resist..

[117] Slettemeas J.S., Anne-Margrete U., Solveig S.M., Gro S.J., Kari G., Madelaine N., Martin S., Marianne S. (2017). Imported food and feed as contributors to the introduction of plasmid-mediated colistin-resistant Enterobacteriaceae to a ‘low prevalence’ country. J. Antimicrob. Chemother..

[118] Eichhorn I., Feudi C., Wang Y., Kaspar H., Feßler A.T., Lü bke-Becker A., Brenner Michael G., Shen J., Schwarz S. (2018). Identification of novel variants of the colistin resistance gene mcr-3 in Aeromonas spp. from the national resistance monitoring programme GERM-Vet and from diagnostic submissions. J. Antimicrob. Chemother..

[119] Ling Z., Yin W., Li H., Zhang Q., Wang X., Wang Z., Ke Y., Wang Y., Shen J. (2017). Chromosome-Mediated mcr-3 Variants in Aeromonas veronii from Chicken Meat. Antimicrob. Agents Chemother..

[120] Lozano-Leon A., Garcia-Omil C., Dalama J., Rodriguez-Souto R., Martinez-Urtaza J., Gonzalez-Escalona N. (2019). Detection of colistin resistance mcr-1 gene in Salmonella enterica serovar Rissen isolated from mussels, Spain, 2012 to2016. Eurosurveillance.

[121] Zhao X., Ye C., Sun C.W. (2017). Serotype Distribution, Antimicrobial Resistance, and Class 1 Integrons Profiles of Salmonella from Animals in Slaughterhouses in Shandong Province, China. Front. Microbiol.

[122] Campos J., Ribeiro S., Vanessa J., Melro C., Vieira L.M., Antunes P. (2016). Screening of novel plasmid-mediated colistin resistance genes *mcr-3*, *mcr-4* and *mcr-5* in *Salmonella* from diverse serotypes and sources. Eur. Congr. Clin. Microbiol. Infect. Dis..

[123] Lv L., Cao Y., Yu P., Huang R., Wang J., Wen Q., Zhi C., Zhang Q., Liu J.-H. (2018). Detection of mcr-1 Gene among *Escherichia coli* Isolates from Farmed Fish and Characterization of mcr-1-Bearing IncP Plasmids. J. Antimicrob. Chemother..

[124] Ghafur A., Shankar C., GnanaSoundari P., Venkatesan M., Mani D., Thirunarayanan M.A., Veeraraghavan B. (2019). Detection of chromosomal and plasmid-mediated mechanisms of colistin resistance in *Escherichia coli* and Klebsiella pneumoniae from Indian food samples. J. Glob. Antimicrob. Resist..

[125] Oliveira C.C., Lopes E.S., Barbosa D.R., Pimenta R.L., Sobrinho N.M.B.A., Coelho S.M.O., Souza M.M.S., Coelho I.S. (2019). Occurrence of the colistin resistance mcr-1 gene in soils from intensive vegetable production and native vegetation. Eur. J. Soil Sci..

[126] Brauer A., Telling K., Laht M., Kalmus P., Lutsar I., Remm M., Kisand V., Tenson T. (2016). Plasmid with Colistin Resistance Gene mcr-1 in Extended-Spectrum-Lactamase-Producing *Escherichia coli* Strains Isolated from Pig Slurry in Estonia. Antimicrob. Agents Chemother..

[127] Zheng B., Huang C., Xu H., Guo L., Zhang J., Wang X., Jiang X., Yu X., Jin L., Li X. (2017). Occurrence and Genomic Characterization of ESBL-Producing, MCR-1-Harboring *Escherichia coli* in Farming Soil. Front. Microbiol..

[128] Li J., Shi X., Yin W., Wang Y., Shen Z., Ding S., Wang S. (2017). A Multiplex SYBR Green Real-Time PCR Assay for the Detection of Three Colistin Resistance Genes from Cultured Bacteria, Feces, and Environment Samples. Front. Microbiol..

[129] Gao Y., Lu C., Shen D., Liu J., Ma Z., Yang B., Ling W., Waigi M.G. (2019). Elimination of the risks of colistin resistance gene (mcr-1) in livestock manure during composting. Environ. Int..

[130] Xia X., Wang Z., Fu Y., Du X., Gao B., Zhou Y., He J., Wang Y., Shen J., Jiang H. (2019). Association of colistin residues and manure treatment with the abundance of mcr-1 gene in swine feedlots. Environ. Int..

[131] Touati M., Hadjadj L., Berrazeg M., Baron S., Rolain J.M. (2019). Emergence of *Escherichia coli* harboring mcr-1 and mcr-3 gene in North West Algerian farmlands. J. Glob. Antimicrob. Resist..

[132] Yanat B., Machuca J., Yahia R.D., Touati A., Pascual Á., Rodríguez-Martínez J.-M. (2016). First report of the plasmid-mediated colistin resistance gene mcr-1 in a clinical *Escherichia coli* isolate in Algeria. Int. J. Antimicrob. Agents.

[133] Jones-Dias D., Manageiro V., Ferreira E., Barreiro P., Vieira L., Moura I.B., Manuela C. (2016). Architecture of Class 1, 2, and 3 Integrons from Gram Negative Bacteria Recovered among Fruits and Vegetables. Front. Microbiol..

[134] Luo J., Yao X., Lv L., Doi Y., Huang X., Huang S., Liu J.-H. (2017). Emergence of mcr-1 in Raoultella ornithinolytica and *Escherichia coli* Isolates from Retail Vegetables in China. Antimicrob. Agents Chemother..

[135] Liu B.T., Li X., Zhang Q., Shan H., Zou M., Song F.J. (2019). Colistin-Resistant mcr-Positive Enterobacteriaceae in Fresh Vegetables, an Increasing Infectious Threat in China. Int. J. Antimicrob. Agents.

[136] Liu B., Song F. (2019). Emergence of two *Escherichia coli* strains co-harboring mcr-1 and bla NDM in fresh vegetables from China. Infect. Drug Resist..

[137] Shen Z., Hu Y., Sun Q., Hu F., Zhou H., Shu L., Ma T., Shen Y., Wang Y., Li J. (2018). Emerging Carriage of NDM-5 and MCR-1 in *Escherichia coli* from Healthy People in Multiple Regions in China: A Cross Sectional Observational Study. EClinicalMedicine.

[138] Yang F., Shen C., Zheng X., Liu Y., El-Sayed Ahmed M.A.E.-G., Zhao Z., Liao K., Shi Y., Guo X., Zhong R. (2019). Plasmid-mediated colistin resistance gene mcr-1 in *Escherichia coli* and Klebsiella pneumoniae isolated from market retail fruits in Guangzhou, China. Infect. Drug Resist..

[139] Mavrici D., Yambao J.C., Lee B.G., Quiñones B., He X. (2017). Screening for the presence of mcr-1/mcr-2 genes in Shiga toxin-producing *Escherichia coli* recovered from a major produce-production region in California. PLoS ONE.

[140] Ruzauskas M., Vaskeviciute L. (2016). Detection of the mcr-1 gene in Escherichia coli prevalent in the migratory bird species Larus argentatus. J. Antimicrob. Chemother..

[141] Oteo J., Mencía A., Bautista V., Pastor N., Lara N., González-González F., García-Peña F.J., Campos J. (2018). Colonization with Enterobacteriaceae-Producing ESBLs, AmpCs, and OXA-48 in Wild Avian Species, Spain 2015–2016. Microb. Drug Resist..

[142] Ngaiganam E.P., Pagnier I., Chaalal W., Leangapichart T., Chabou S., Rolain J.M., Diene S.M. (2019). Investigation of urban birds as source of β-lactamase-producing Gram-negative bacteria in Marseille city, France. Acta Vet. Scand..

[143] Sellera F.P., Fernandes M.R., Sartori L., Carvalho M.P.N., Esposito F., Nascimento C.L., Dutra G.H.P., Mamizuka E.M., Pérez-Chaparro P.J., Mcculloch J.A. (2017). *Escherichia coli* carrying IncX4 plasmid-mediated mcr-1 and bla CTX-M genes in infected migratory *Magellanic penguins* (*Spheniscus magellanicus*). J. Antimicrob. Chemother..

[144] Liakopoulos A., Mevius D.J., Olsen B., Bonnedahl J. (2016). The colistin resistance mcr-1 gene is going wild. J. Antimicrob. Chemother. Chemother.

[145] Mohsin M., Raza S., Roschanski N., Schaufler K., Guenther S. (2016). First description of plasmid-mediated colistin-resistant extended-spectrum β-lactamase-producing *Escherichia coli* in a wild migratory bird from Asia. Int. J. Antimicrob. Agents.

[146] Zou H., Zheng B., Sun M., Ottoson J., Li Y., Berglund B., Chi X., Ji X., Li X., Stålsby Lundborg C. (2019). Evaluating Dissemination Mechanisms of Antibiotic-Resistant Bacteria in Rural Environments in China by Using CTX-M-Producing *Escherichia coli* as an Indicator. Microb. Drug Resist..

[147] Hadjadj L., Riziki T., Zhu Y., Li J., Diene S., Rolain J.-M., Hadjadj L., Riziki T., Zhu Y., Li J. (2017). Study of mcr-1 Gene-Mediated Colistin Resistance in Enterobacteriaceae Isolated from Humans and Animals in Different Countries. Genes (Basel).

[148] Swift B.M.C., Bennett M., Waller K., Dodd C., Murray A., Gomes R.L., Humphreys B., Hobman J.L., Jones M.A., Whitlock S.E. (2019). Anthropogenic environmental drivers of antimicrobial resistance in wildlife. Sci. Total Environ..

[149] Wang X., Wang Y., Wang Y., Zhang S., Shen Z., Wang S. (2018). Emergence of the colistin resistance gene mcr-1 and its variant in several uncommon species of Enterobacteriaceae from commercial poultry farm surrounding environments. Vet. Microbiol..

[150] Zhang J., Wang J., Chen L., Kamal Yassin A., Kelly P., Butaye P., Li J., Gong J., Cattley R., Qi K. (2018). Housefly (*Musca domestica*) and Blow Fly (*Protophormia terraenovae*) as Vectors of Bacteria Carrying Colistin Resistance Genes. Appl. Environ. Microbiol..

[151] Fukuda A., Usui M., Okubo T., Tagaki C., Sukpanyatham N., Tamura Y. (2018). Co-harboring of cephalosporin (bla)/colistin (mcr) resistance genes among Enterobacteriaceae from flies in Thailand. FEMS Microbiol. Lett..

[152] Yang Q.E., Tansawai U., Andrey D.O., Wang S., Wang Y., Sands K., Kiddee A., Assawatheptawee K., Bunchu N., Hassan B. (2019). Environmental dissemination of mcr-1 positive Enterobacteriaceae by *Chrysomya* spp. (common blowfly): An increasing public health risk. Environ. Int..

[153] Liu P.-Y., Weng L.-L., Tseng S.-Y., Huang C.-C., Cheng C.-C., Mao Y.-C., Tung K.-C. (2017). Colistin Resistance of Pseudomonas aeruginosa Isolated from Snakes in Taiwan. Can. J. Infect. Dis. Med. Microbiol..

[154] Teo J.W., Chew K.L., Lin R.T. (2016). Transmissible colistin resistance encoded by mcr-1 detected in clinical Enterobacteriaceae isolates in Singapore. Emerg. Microbes Infect. ISSN.

[155] Millan A.S., Peña-Miller R., Toll-Riera M., Halbert Z.V., Mclean A.R., Cooper B.S., Maclean R.C. (2014). Positive selection and compensatory adaptation interact to stabilize non-transmissible plasmids. Nat. Commun..

[156] Wu R., Yi L., Yu L., Wang J., Liu Y., Chen X., Lv L., Yang J., Liu J.-H. (2018). Fitness Advantage of mcr-1–Bearing IncI2 and IncX4 Plasmids in Vitro. Front. Microbiol..

[157] Shen Y., Zhou H., Xu J., Wang Y., Zhang Q., Walsh T.R., Shao B., Wu C., Hu Y., Yang L. (2018). Anthropogenic and environmental factors associated with high incidence of mcr-1 carriage in humans across China. Nat. Microbiol..

[158] Walsh T.R., Wu Y. (2016). China bans colistin as a feed additive for animals. Lancet Infect. Dis..

